# Applications of newly defined diamond Pythagorean fuzzy CODAS method via multi-criteria decision-making problems

**DOI:** 10.1371/journal.pone.0325018

**Published:** 2025-12-12

**Authors:** Muhammad Bilal Khan, Miguel Vivas Cortez, Bandar Bin-Mohsin, Loredana Ciurdariu, Nurnadiah Zamri

**Affiliations:** 1 Department of Mathematics and Computer Science, Transilvania University of Brasov, Brasov, Romania; 2 Faculty of Informatics and Computing, Universiti Sultan Zainal Abidin, Terengganu, Malaysia; 3 Escuela de Ciencias Físicas y Matemáticas, Facultad de Ciencias Exactas y Naturales, Pontificia Universidad Católica del Ecuador, Apartado, Quito, Ecuador; 4 Department of Mathematics, College of Science, King Saud University, Riyadh, Saudi Arabia; 5 Department of Mathematics, Politehnica University of Timisoara, Timisoara, Romania; Ankara Yildirim Beyazit University / Worcester Polytechnic Institute, TÜRKIYE

## Abstract

The diverse decision values may fail to capture an accurate perspective when multiple decision-makers are part of the process. To address this challenge, this work introduces the diamond Pythagorean fuzzy set (Dia‑*PyFS*), an advancement over both the Pythagorean fuzzy (*PyFS*) set and the interval-valued Pythagorean fuzzy set (*IVPyFS*). Due to the established extension of intuitionistic fuzzy sets into Pythagorean fuzzy sets, the Dia‑*PyFS* model, a broader form of the diamond intuitionistic fuzzy set model, demonstrates enhanced performance. We then introduce the Dia‑*PyFS* as an extension of *PyFS*. The Diamond Pythagorean fuzzy values that define the elements within Dia‑*PyFS* may share a common norm. For Dia‑*PyFS*, we define fundamental algebraic and arithmetic operations, including union, intersection, addition, multiplication, and scalar multiplication, and analyze their primary properties. Additionally, we propose some new Dia‑*PyF* weighted average and geometric aggregation operators as well as explore their unique properties. We also then propose several algebraic operations between Dia‑*PyFVs* using general triangular *𝓉*-norms and *𝓉*-conorms. To transform input values represented by Dia‑*PyFs* into a single output value, we also introduce specific weighted aggregation operators based on these algebraic methods. Additionally, the Dia‑*PyFS* framework builds upon the “combinative distance-based assess” (*CODAS*) methodology, which relies on both Euclidean and Hamming distances. To illustrate the applicability of this new approach, the feasibility and suitability of the Dia‑*PyFS* set approach for choosing the best options are demonstrated by the summary and comparative analysis of the produced reports.

## 1. Introduction

The concept of fuzzy sets (*FS*) was introduced through a function, often referred to as a membership function, which assigns a numerical value between 0 and 1 to represent the degree of an element’s membership. This approach has been applied to manage the uncertainty present in real-world scenarios. Zadeh’s theory of fuzzy sets [1] has proven effective in addressing various types of uncertainty. To better represent uncertainty, many researchers have explored this concept extensively. Later, Atanassov [2] proposed intuitionistic fuzzy sets (*IFSs*) as an extension of fuzzy sets, incorporating both membership and non-membership degrees. For more information, see [3–10] and the references therein.

Yager [11] introduced Pythagorean fuzzy sets (*PyFS*s) as an effective tool in multi-criteria decision-making (*MCDM*) to address a broader array of situations involving uncertainty. This concept extends intuitionistic fuzzy sets (*IFS*), with membership and non-membership degrees that satisfy the condition that the sum of their squares does not exceed 1. Due to its quadratic structure, a *PyFS* can widen the variability range for membership and non-membership degrees to the unit circle, thereby enhancing its capacity to represent uncertainty compared to an *IFS*.

Different types of *FSs* can be analyzed using points, point pairs, or point triples within a closed interval [12]. This approach requires experts to assign precise numerical values, enhancing the accuracy of the decision-making process. To address the limitations of intuitionistic fuzzy sets (*IFS*), Atanassov [13] introduced circular intuitionistic fuzzy sets (Cir-*IFS*s). In Cir-*IFS*s, the circular form represents the uncertainty within membership and non-membership degrees. Each element's membership and non-membership in a Cir-*IFS* is depicted using a circular structure, where two non-negative real numbers indicate the circle’s center, with their sum restricted to a maximum of 1. Cir- *IFS*s facilitate the expression of uncertainty by enabling a flexible adjustment of membership and non-membership degrees. For more information, see [14–23] and the references therein.

Bozyigit et al. [24] recently introduced the concept of circular Pythagorean fuzzy sets (Cir-*PyFSs*) and circular Pythagorean fuzzy values (*Cir*-*PyFV*). This novel approach is a comprehensive extension of Pythagorean fuzzy sets (*PyFSs*) and circular intuitionistic fuzzy sets (Cir-*IFS*s). In Cir-*PyFS*, membership and non-membership degrees are depicted using a circular representation. The circular center consists of non-negative real values uuu and vvv, constrained such that the sum of their squares does not exceed 1. Due to this unique structure, Cir-*PyFS* effectively models information with circular points defined by a specific center and radius, enhancing its capacity to capture the uncertainty of data. As a result, Cir-*PyFS* allows experts to evaluate options within a broader and more flexible framework, facilitating the development of more nuanced and complex decisions. For further information, related to *IFS*s and *PyFS*, see [25–32] and the references therein.

*MCDM* approaches are widely used across various scientific disciplines [33–35]. These approaches are categorized into two main types: human-based methods (such as the best-worst method and the analytic hierarchy process [AHP]) and mathematical methods (including TOPSIS and VIKOR) [36,37]. Mathematical techniques face some limitations: (i) one key aspect in decision-making research is the normalization process, which standardizes different evaluation scales by converting them into dimensionless values. To reach a conclusive result, researchers apply various data normalization methods, such as vector, linear, and linear–max–min normalization. Each normalization technique produces unique scales that influence data behavior, ultimately affecting the final decision [38]. (ii) In the TOPSIS method, the maximum and minimum values are used to define positive and negative ideal solutions. However, certain limitations might affect the general applicability of this approach. For instance, the ideal blood pressure range is typically found between the highest and lowest observed values [39]. (iii) Mathematical methods cannot precisely determine the weights of evaluation criteria. Consequently, an external approach, such as human judgment or prioritization methods, is necessary to assign these weights [40].

Human-based techniques have certain limitations, including: (i) Inconsistencies in factor weighting due to paired comparisons, which is a key issue in human-based methods [41]. For the priorities determined by AHP to be reliable, the pairwise comparison matrices must pass a consistency check. In these matrices, decision-makers assign numerical values to represent paired comparisons based on their expertise and knowledge [42]. However, due to the complexity of the decision problem and possible gaps in experience or knowledge, contradictions can arise within these matrices. When comparing factors, decision-makers must indicate how strongly one factor outweighs another, typically on a scale from 1 to 9, [43]. (ii) The process of subjective comparisons is unusual, making the task mentally demanding. In other words, comparing two unrelated factors poses a challenge as it is not an intuitive process [39]. (iii) Additionally, the method becomes more complex due to the considerable time required for conducting pairwise and reference comparisons across numerous criteria [44].

Moreover, both human and mathematical methods have limitations in managing data ambiguity and imprecision [45,46]. The FWZIC method, developed by [47], has shown superior performance over other human-based *MCDM* methods due to its unique capability of calculating criteria weights with 0% inconsistency. However, the original FWZIC approach had limitations in addressing the imprecision and uncertainty caused by expert hesitation. FWZIC has since been adapted for various fuzzy contexts—including neutrosophic fuzzy sets, Pythagorean fuzzy sets, cubic *PyFS*, interval type-2 trapezoidal fuzzy sets, dual-hesitant fuzzy sets, q-rung orthopair fuzzy sets, T-spherical fuzzy sets, and Pythagorean m-polar fuzzy sets—to better handle issues involving ambiguity and imprecision [41–48]. Despite significant efforts, the problem of ambiguity and imprecision in data persists.

An effective solution is essential to comprehensively tackle all the challenges outlined above. This requires the development of a new *MCDM* mathematical method capable of reducing the number of comparisons, eliminating the need for normalization, and successfully incorporating the concept of an ideal solution. The approach should focus on creating implicit and fair comparisons, avoiding forced or unnatural comparisons, preventing inconsistencies, and minimizing the use of complex mathematical computations. Additionally, the proposed method must account for informational ambiguity. To address these challenges, this study aims to introduce an innovative *MCDM* technique that leverages the concept of an ideal solution and an opinion matrix. The proposed method will produce rational and coherent decisions, drawing on the insights of the decision-makers.

Recognizing and integrating the diverse needs, interests, and expectations of citizens presents a significant challenge in the execution of SSL projects [49]. Ensuring that the solutions align with the community’s values and objectives is vital, as it directly impacts citizen acceptance. In this context, city planners and decision-makers may find *MCDM* to be a valuable approach for assessing the relationship between smart cities and public approval. This method can assist in making well-informed decisions during the planning and execution of successful smart city initiatives [40].

Although *MCDM* methods such as VIKOR (VlseKriterijuska Optimizacija I Komoromisno Resenje) and TOPSIS (Technique for Order of Preference by Similarity to Ideal Solution) [50,51] are effective for ranking alternatives, they are insufficient for determining the exact grade [52,53]. These approaches do not account for the decision threshold or probability, even though they consider the distance between alternatives and ideal solutions [54]. However, three-way decisions (TWDs) could offer a solution to this issue. Additionally, these *MCDM* methods have limitations, such as the need for normalization, the number of comparisons, inconsistencies, forced comparisons, the extent of mathematical computations, and the presence of informational ambiguity [55]. Recently, Khan et. al [56] introduced the concept of diamond intuitionistic fuzzy sets (Dia‑*IFS*s). Additionally, some new basic operations and relations are derived then developed Dia‑*IFS*s via *IFS*. With the support relation, an illustrated medical diagnosis example is provided as the application of Dia‑*IFS*s. For further study related to *𝓉*-norms and *𝓉*-conorms, see [57–63] and the references therein. Al-shami et al., [64] and Al-shami et al., [65] introduced SR‐Fuzzy Sets and (2, 1)-Fuzzy sets as the generalization fuzzy sets. Note that these fuzzy sets applications are more effective than other classical fuzzy sets. Similarly, Thakur et al. [66] another new version of fuzzy sets that give more freedom to decision makers to find the membership and non-membership values. Additionally, the application theses fuzzy sets can found at large scale indifferent fields of our life.

To address the challenges associated with the selection and evaluation of electric auto rickshaw frameworks, we propose a novel *CODAS* method that integrates *MCDM* within the framework of newly defined diamond-Pythagorean fuzzy sets (Dia‑*PyFSs*) as well as related aggregation operators. This paper introduce Dia‑*PyFSs* and the *CODAS* method, which employs distance measure based on opinion scores in the context of Dia‑*PyFSs*. The aim of this approach is to compute the Hamming and Euclidean distance formulas for each alternative in decision-making scenarios. Furthermore, decision thresholds for each option are established using Bayesian decision theory within the Dia‑*PyF* environment. Additionally, the relative assessment matrix method is utilized via assessment scores in the Dia‑*PyFS* context to prioritize the criteria influencing the selection and assessment of electric auto rickshaw frameworks.

The key benefits of employing *MCDM* for grading alternatives with the *CODAS* method are summarized as follows: The proposed approach demonstrates effectiveness in managing information related to dynamic and uncertain scenarios. Moreover, by utilizing *MCDM*, it can transform conventional ranking outcomes into objective classification results. The method also offers flexibility by adjusting the risk avoidance coefficient, enabling it to dynamically incorporate situational data. Based on these features, the following highlights the main innovations and contributions of this paper:

- The ideas of Dia-*PyFS* and Dia-*PyFV* are introduced in this study.
- A technique for converting a set of *PyFV* into Dia-*PyFS* is obtained, the multi-attribute decision making *MCDM* can be resolved in this manner.
- Dia-shape indicate an element's membership or non-membership in a Dia-*PyFS*. Its structure allows for more sensitive modeling in the continuous environment using *MCDM* theory.
- For *Dia*-*PyFS*, certain algebraic operations are defined using *𝓉*-norms and *𝓉*-conorms.
- Some weighted arithmetic and geometric aggregation operators are supplied with the support of these operations.
- To support our proposed methodology, this paper includes illustrated *MCDM* problem via *CODAS* method.

The structure of the remaining sections is as follows: Section 2 provides a review of the most pertinent literature. Section 3 outlines the core concepts of Dia-*PyFSs* and Dia-PyFVs. Come properties of *Dia*-*PyFV*s are presented and analyzed via basic operations as well as a techniques is obtained to develop *Dia*-*PyFSs* via *PyFSs* in Section 4. Section 5 characterize the basic operation using triangular *𝓉*-norms and triangular *𝓉*-conorms. In Section 6, some aggregation operators are obtained. Additionally, some exceptional cases are discussed. Additionally, some distance measures are introduced by using *Dia*-*PyFSs*. In Section 7, steps of algorithm for *MCDM* technique is discussed using *CODAS* method. The proposed methodology is applied to a problem concerning the selection of the optimal solution over electric auto rickshaw framework. Finally, Section 8 offers the conclusion of the paper.

## 2. Preliminaries

This section explains several fundamental concepts utilized in this study.

**Definition 1.** ([11]) Let us have a fixed universe *E* and its sub-set P. The set


$$P=\left\{\left\langle \rho ,\;{\text{ɀ}}_{P}(\rho ),\;{\text{ɤ}}_{P}(\rho )\right\rangle \;:\text{for}\;\text{all}\;\rho \in E\right\},$$

where $\text{0}\;\leq {\left({\text{ɀ}}_{P}(\rho )\right)}^{\text{2}}+{\left({\text{ɤ}}_{P}(\rho )\right)}^{\text{2}}\leq \text{1},\;$ is called *PyFS* and functions ${\text{ɀ}}_{P},{\text{ɤ}}_{P}\;:E\to [\text{0},\;\text{1}]$ indicate the degree of membership (validity, etc.) and non-membership (non-validity, etc.) of element ρ∈E to a fixed set P⊆E. Now, we can define also function $\pi_{P}:E\;\to \;[\text{0},\;\text{1}]$ by means of


$$\pi_{P}(\rho )=\;\sqrt{\text{1}-{\left({\text{ɀ}}_{P}(\rho )\right)}^{\text{2}}-{\left({\text{ɤ}}_{P}(\rho )\right)}^{\text{2}}}$$

and it corresponds to degree of indeterminacy (uncertainty, etc.). An *PyFV* is the pair “$<{\text{ɀ}}_{P}(\rho ),{\text{ɤ}}_{P}(\rho )>$” given an element ρ of *X*. To make things easier to understand, we can write $\tilde{\text{𝓅}}=<{\text{ɀ}}_{\tilde{\text{𝓅}}},{\text{ɤ}}_{\tilde{\text{𝓅}}}>$, where ${\text{ɀ}}_{\tilde{\text{𝓅}}}\in [\text{0},\text{1}]$, ${\text{ɤ}}_{\tilde{\text{𝓅}}}\in [\text{0},\text{1}]$ and $\text{0}\leq {\text{ɀ}}_{\tilde{\text{𝓅}}}+{\text{ɤ}}_{\tilde{\text{𝓅}}}\leq \text{1}$. The degree of indeterminacy is represented by $\pi_{\tilde{\text{𝓅}}}$, subject to the constraints that $\pi_{\tilde{\text{𝓅}}}\in [\text{0},\text{1}]$ and $\pi_{\tilde{\text{𝓅}}}=\text{1}-{\text{ɀ}}_{\tilde{\text{𝓅}}}-{\text{ɤ}}_{\tilde{\text{𝓅}}}$.

The definition of the complement of an *IFV* $\tilde{\text{𝓅}}=<{\text{ɤ}}_{\tilde{\text{𝓅}}},{\text{ɀ}}_{\tilde{\text{𝓅}}},\pi_{\tilde{\text{𝓅}}}>$ is as follows:


$${\tilde{\text{𝓅}}}^{C}=<{\text{ɤ}}_{\tilde{\text{𝓅}}},{\text{ɀ}}_{\tilde{\text{𝓅}}},\pi_{\tilde{\text{𝓅}}}>.$$

**Definition 2.** ([56]) Let us have a fixed universe *E* and its sub-set P. The set


$${\text{Ϣ}}_{ℵ}=\;\{\left\langle \rho ,\;\text{ɀ}(\rho ),\;\text{ɤ}(\rho );ℵ\;\right\rangle |\;\rho \in E\},$$

where 0 ≤ɀ(ρ)+ɤ(ρ)≤1 and ℵ∈[0,2 ] is called Dia-*IFS* and functions ɀ,ɤ :E→[0, 1] indicate the degree of membership (validity, etc.) and non-membership (non-validity, etc.) of element ρ∈E to a fixed set P⊆E. Now, we can define also function π:E → [0, 1] by means of


**Fig 1:**
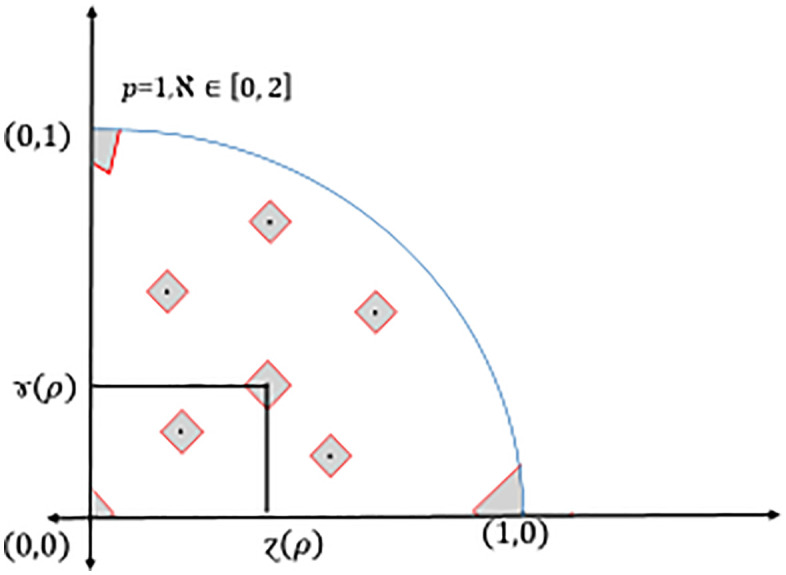
Geometrical presentation of Dia-PyFS.

π(ρ)=1−ɀ(ρ)+ɤ(ρ).

and it corresponds to degree of indeterminacy (uncertainty, etc.), see Fig 1.

On the other hand ${\text{Ϣ}}_{ℵ}^{py}$ can also be defined by using following approach such that.

Let ${\text{Ϣ}}_{\text{1}}=\left\{\left\langle \alpha ,\beta \right\rangle \;:\alpha ,\beta \in [\text{0},\;\text{1}],\text{and}\;\alpha +\beta \leq \text{1}\right\}$. Then,


$${\text{Ϣ}}_{ℵ}\;=\;\left\{\left\langle \rho ,\;{ℵ}_{\;}^{\text{1}}\left(\text{ɀ}(\rho ),\;\text{ɤ}(\rho )\right)\right\rangle \;:\rho \in E\right\},$$

Where


$$\begin{array}{cc} {ℵ}_{\;}^{\text{1}}\left(\text{ɀ}(\rho ),\;\text{ɤ}(\rho )\right) & =\left\{\left\langle \alpha ,\beta \right\rangle \;:\alpha ,\beta \in [\text{0},\;\text{1}]\;\text{and}\;\left(\left|\text{ɀ}(\rho )-\alpha \right|+\left|\text{ɤ}(\rho )-\beta \right|\right)\leq ℵ\right\}\cap {\text{Ϣ}}_{\text{1}}\\ & =\left\{\left\langle \alpha ,\beta \right\rangle \;:\alpha ,\beta \in [\text{0},\;\text{1}],\;\left(\left|\text{ɀ}(\rho )-\alpha \right|+\left|\text{ɤ}(\rho )-\beta \right|\right)\leq ℵ\;\text{and}\;\alpha +\beta \leq \text{1}\right\}. \end{array}$$

Menger's ([58]) notion of probabilistic metric spaces served as the inspiration for Schweizer and Sklar's [41] introduction of the ideas of *𝓉*-norm and *𝓉*-conorm. In statistics and decision-making, these ideas are essential. The closed unit interval is the basis for the binary operations known as *𝓉*-norm and *𝓉*-conorms in algebra.

**Definition 3.** ([59,60]) A mapping T:[0,1]×[0,1]→[0,1] that satisfies the following characteristics is called a *𝓉*-norm:

(T1) Border condition: T(α,1)=α for all α∈[0,1],

(T2) Commutativity: T(α,β)=T(β,α) for all α,β∈[0,1],

(T3) Associativity: T(α,T(β,ɤ))=T(T(α,β),ɤ) for all α,β,ɤ∈[0,1],

(T4) Monotonicity: $T(\alpha ,\beta )\leq T\left(\alpha^{\prime },\beta^{\prime }\right)$ whenever $\alpha \leq \alpha^{\prime }$ and $\beta \leq \beta^{\prime }$ for all $\alpha ,\alpha^{\prime },\beta ,\beta^{\prime }\in [\text{0},\text{1}]$.

**Definition 4.** ([59,60]) A mapping S:[0,1]×[0,1]→[0,1] that satisfies the following characteristics is called a *𝓉*-conorm:

(S1) Border condition: S(α,0)=α for all α∈[0,1] (border condition),

(S2) Commutativity: S(α,β)=S(β,α) for all α,β∈[0,1] (commutativity),

(S3) Associativity: S(α,S(β,ɤ))=S(S(α,β),ɤ) for all α,β,ɤ∈[0,1] (associativity),

(S4) Monotonicity: $S(\alpha ,\beta )\leq S(\alpha^{\prime },\beta^{\prime }\;$) whenever $\alpha \leq \alpha^{\prime }$ and $\beta \leq \beta^{\prime }$ for all $\alpha ,\alpha^{\prime },\beta ,\beta^{\prime }\in [\text{0},\text{1}]$ (monotonicity).

**Definition 5.** ([60,61]) A function 𝒷:[0,1]→ [0,∞] with 𝒷(1)=0 that is strictly decreasing and satisfies 𝒷(1)=0 is referred to as the additive generator of a *𝓉*-norm T if the relationship $T(\alpha ,\beta )={\text{𝒷}}^{-\text{1}}(\text{𝒷}(\alpha )+\text{𝒷}(\beta ))$ holds for all (α,β)∈[0,1]×[0,1].

The concept of a fuzzy complement is required to determine the additive generator of a dual *𝓉*-conorm defined on the interval [0,1].

**Definition 6.** ([3]) A fuzzy complement is a function N:[0,1]→[0,1] that meets the following criteria:

(N1) N(0)=1 and N(1)=0 (boundary conditions),
(N2) N(α)≥N(β) whenever α≤β for all α,β∈[0,1] (monotonicity),
(N3) Continuity,
(N4) N(N(α))=α for all α∈[0,1] (involution).

The function N:[0,1]→[0,1] given by $N(\alpha )={\left(\text{1}-\alpha^{p}\right)}^{\text{1}/p}$, where p∈(0,∞), represents a fuzzy complement. When p=1,N simplifies to the Pythagorean fuzzy complement $N(\alpha )={\left(\text{1}-\alpha^{\text{2}}\right)}^{\text{1}/\text{2}}$.

**Definition 7.** ([63]) Let T be a *𝓉*-norm and S be a *𝓉*-conorm on the interval [0, 1]. If T(α,β)=N(S(N(α),N(β))) and S(α,β)=N(T(N(α),N(β))), then T and S are referred to as dual with respect to the fuzzy complement *N*.

**Remark 1.** Let T represent a *𝓉*-norm on the interval [0,1]. The corresponding dual *𝓉*-conorm S with regard to the intuitionistic fuzzy complement *N* is defined as follows:


S(α,β)=1−T(1−α,1−β)

It is important to mention that T qualifies as an Archimedean *𝓉*-norm if and only if T(α,α)<α for all α∈(0,1), while S is classified as an Archimedean *𝓉*-conorm if and only if S(α,α)>α [60,61]. Klement et al. [62] demonstrated that continuous Archimedean *𝓉*-norms can be represented through their additive generators, as established in the following theorem.

**Theorem 1** ([62]). Let T represent a *𝓉*-norm on [0, 1]. The following statements are equivalent:

(i) T is a continuous Archimedean *𝓉*-norm.
(ii) T possesses a continuous additive generator, meaning there exists a continuous, strictly decreasing function 𝒷:[0,1]→[0,∞] with 𝓉(1)=0 such that $T(\alpha ,\beta )={\text{𝒷}}^{-\text{1}}(\text{𝒷}(\alpha )+\text{𝒷}(\beta ))$ for all (α,β)∈[0,1]×[0,1].

## 3. Diamond Pythagorean fuzzy sets

This section covers some fundamental properties of the diamond Pythagorean fuzzy set sets utilized in this work.

**Fig 2:**
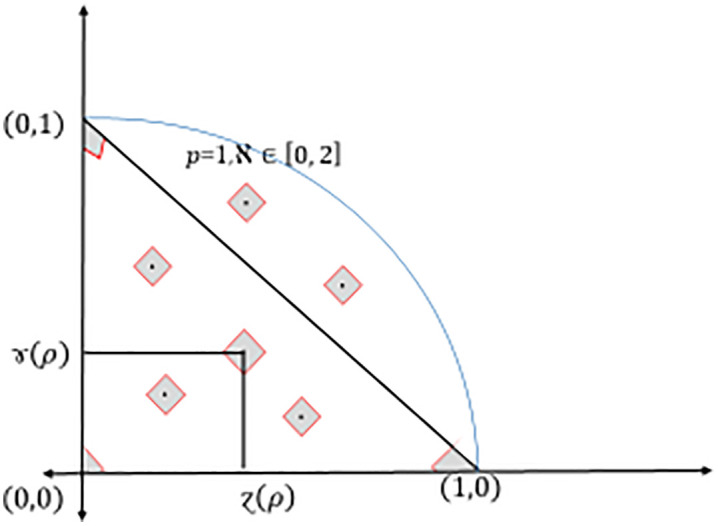
Comparison between Dia-*IFS* and Dia-*PyFS.*

**Definition 8.** Let us have a fixed universe *E* and its sub-set P. The set


$${\text{Ϣ}}_{ℵ}^{py}=\;\{\left\langle \rho ,\;\text{ɀ}(\rho ),\;\text{ɤ}(\rho );ℵ\;\right\rangle |\;\rho \in E\},$$

where $\text{0}\;\leq {\left(\text{ɀ}(\rho )\right)}^{\text{2}}+{\left(\text{ɤ}(\rho )\right)}^{\text{2}}\leq \text{1}$ and ℵ∈[0,2 ] is called Dia‑*PyFS* and functions ɀ,ɤ :E→[0, 1] indicate the degree of membership (validity, etc.) and non-membership (non-validity, etc.) of element ρ∈E to a fixed set P⊆E. Now, we can define also function π:E → [0, 1] by means of


$$\pi (\rho )=\sqrt{\text{1}-{\left(\text{ɀ}(\rho )\right)}^{\text{2}}-{\left(\text{ɤ}(\rho )\right)}^{\text{2}}}.$$

and it corresponds to degree of indeterminacy (uncertainty, etc.), see Figs 1 and 2.

On the other hand ${\text{Ϣ}}_{ℵ}^{py}$ can also be defined by using following approach such that.

Let ${\text{Ϣ}}_{\text{1}}=\left\{\left\langle \alpha ,\beta \right\rangle \;:\alpha ,\beta \in [\text{0},\;\text{1}],\text{and}\;\alpha^{\text{2}}+\beta^{\text{2}}\leq \text{1}\right\}$. Then,


$${\text{Ϣ}}_{ℵ}^{py}\;=\;\left\{\left\langle \rho ,\;{ℵ}_{\;}^{\text{1}}\left(\text{ɀ}(\rho ),\;\text{ɤ}(\rho )\right)\right\rangle \;:\rho \in E\right\},$$

Where


$${ℵ}_{\;}^{\text{1}}\left(\text{ɀ}(\rho ),\;\text{ɤ}(\rho )\right)=\left\{\left\langle \alpha ,\beta \right\rangle \;:\alpha ,\beta \in [\text{0},\;\text{1}\text{and}\;\left(\left|\text{ɀ}(\rho )-\alpha \right|+\left|\text{ɤ}(\rho )-\beta \right|\right)\leq ℵ\right\}\cap {\text{Ϣ}}_{\text{1}},$$

$$=\left\{\left\langle \alpha ,\beta \right\rangle \;:\alpha ,\beta \in [\text{0},\;\text{1}],\;\left(\left|\text{ɀ}(\rho )-\alpha \right|+\left|\text{ɤ}(\rho )-\beta \right|\right)\leq ℵ\;\text{and}\;\alpha^{\text{2}}+\beta^{\text{2}}\leq \text{1}\right\}.$$

**Fig 3:**
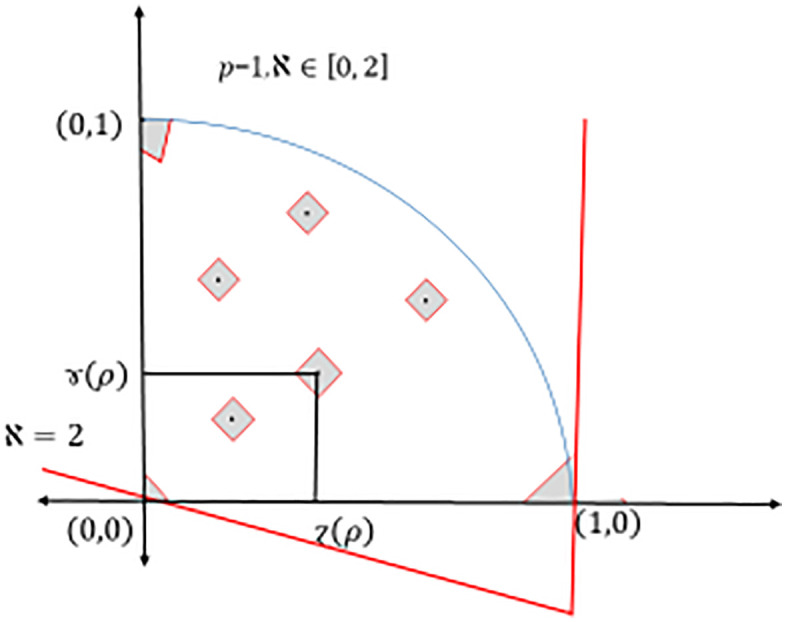
Triangular coverage of different ℵ values of Dia-*PyFS.*

Note that, if we want to cover the diamond-Pythagorean fuzzy interpretation triangle, then ℵ∈[0, 2], see Fig 3.

Here is the restriction of Definition 8; however, in this case, the Pythagorean fuzzy interpretation triangle cannot be fully covered.

**Fig 4:**
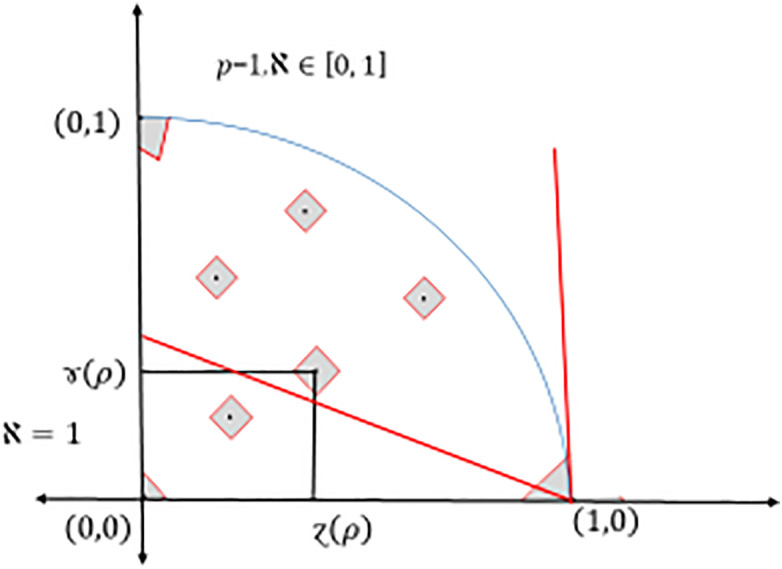
Triangular coverage of different ℵ values of Dia-*PyFS.*

**Definition 9.** Let us have a fixed universe *E* and its sub-set P. The set


$${\text{Ϣ}}_{ℵ}^{py}=\;\{\left\langle \rho ,\;\text{ɀ}(\rho ),\;\text{ɤ}(\rho );ℵ\;\right\rangle |\;\rho \in E\},$$

where $\text{0}\;\leq {\left(\text{ɀ}(\rho )\right)}^{\text{2}}+{\left(\text{ɤ}(\rho )\right)}^{\text{2}}\leq \text{1}$ and ℵ∈[0,1]  with p≥1 is called Dia-*PyFS* and functions ɀ,ɤ :E→[0, 1] indicate the degree of membership (validity, etc.) and non-membership (non-validity, etc.) of element ρ∈E to a fixed set P⊆E. Now, we can define also function π:E → [0, 1] by means of


$$\pi (\rho )=\sqrt{\text{1}-{\left(\text{ɀ}(\rho )\right)}^{\text{2}}-{\left(\text{ɤ}(\rho )\right)}^{\text{2}}},$$

and it corresponds to degree of indeterminacy (uncertainty, etc.), see again Figs 1–2 and 4.

### 3.1. Development of diamond Pythagorean fuzzy sets via Pythagorean fuzzy sets

In this section, we will discuss the procedure of calculating the Dia‑*PyFS* in order to convert *PyFS* to Dia‑*PyFS*.

Assume that there are Pythagorean fuzzy pairs in an *PyFS* ${\text{Ϣ}}_{{ℵ}_{i}}^{py}$ with the following shapes: $\left\{\;\left({\text{ɀ}}_{i,\text{1}},\;{\text{ɤ}}_{i,\text{1}}\right),\;\left({\text{ɀ}}_{i,\text{2}},\;{\text{ɤ}}_{i,\text{2}}\right),\;\left({\text{ɀ}}_{i,\text{3}},\;{\text{ɤ}}_{i,\text{3}}\right),\ldots \ldots \right\}$, where *m* is a numerical value of an PyFS ${\text{Ϣ}}_{{ℵ}_{i}}^{py}$ that contains *n*_*i*_, the number of Pythagorean fuzzy pairs with ${\text{Ϣ}}_{{ℵ}_{i}}^{py}$.

The following formula can be used to determine the “arithmetic average” of diamond Pythagorean fuzzy pairs:


$$\left(\text{ɀ}\left({\text{Ϣ}}_{{ℵ}_{i}}^{py}\right),\text{ɤ}\left({\text{Ϣ}}_{{ℵ}_{i}}^{py}\right)\right)=\left(\sum_{j=\text{1}}^{n_{i}}\frac{{\left({\text{ɀ}}_{i,j}\right)}^{\text{2}}}{n_{i}},\sum_{j=\text{1}}^{n_{i}}\frac{{\left({\text{ɤ}}_{i,j}\right)}^{\text{2}}}{n_{i}}\right).$$

The ℵ of $\left(\text{ɀ}\left({\text{Ϣ}}_{{ℵ}_{i}}^{py}\right),\text{ɤ}\left({\text{Ϣ}}_{{ℵ}_{i}}^{py}\right)\right)$ has the maximum Euclidean distance value.


$${ℵ}_{i}=\underset{\text{1}\leq j\leq n_{i}}{\text{𝓂}\text{𝒶}\text{𝓍}}\left(\left(\left|\text{ɀ}\left({\text{Ϣ}}_{{ℵ}_{i}}^{py}\right)-{\text{ɀ}}_{i,j}\right|+\left|\text{ɤ}\left({\text{Ϣ}}_{{ℵ}_{i}}^{py}\right)-{\text{ɤ}}_{i,j}\right|\right)\right).$$

thus, *PyFS* is being changed into Dia‑*PyFS*.

**Definition 10.** A diamond Pythagorean fuzzy set is a collection of


$${\text{Ϣ}}_{ℵ}^{py}=(\text{ɀ},\text{ɤ};ℵ),$$

where ${\text{Ϣ}}_{ℵ}^{py}$ represent the diamond Pythagorean fuzzy value with conditions;

(i) $\text{0}\leq {\left(\text{ɀ}(x)\right)}^{\text{2}}+{\left(\text{ɤ}(x)\right)}^{\text{2}}\leq \text{1}$, (ii) 0≤ɀ(x),ɤ(x)≤1, (ii) 0≤ℵ≤2.

For the sake of simplicity, the set of diamond Pythagorean fuzzy value (Dia‑*PyFVs*).

In order to facilitate collective decision-making, we now create a mechanism for converting collections of *PyFS*s into a Dia‑*PyFVs*.

**Proposition 1.** Let a set of *PyFVs* be denoted as $\left\{{\text{Ϣ}}_{{ℵ}_{\text{1}}}^{py}=\left({\text{ɀ}}_{\text{1}},\;{\text{ɤ}}_{\text{1}};{ℵ}_{\text{1}}\right),\;{\text{Ϣ}}_{{ℵ}_{\text{2}}}^{py}=\left({\text{ɀ}}_{\text{2}},\;{\text{ɤ}}_{\text{2}};{ℵ}_{\text{2}}\right),\ldots \ldots \ldots ,\;{\text{Ϣ}}_{{ℵ}_{n}}^{py}=\left({\text{ɀ}}_{n},\;{\text{ɤ}}_{n};{ℵ}_{n}\right)\right\}$. Then


$${\text{Ϣ}}_{ℵ}^{py}=\;\left\langle \;\text{ɀ},\;\text{ɤ};ℵ\;\right\rangle ,$$

is a Dia‑*PyFV* with

$\text{ɀ}=\sum_{j=\text{1}}^{n_{i}}\frac{{\left({\text{ɀ}}_{i,j}\right)}^{\text{2}}}{n_{i}}$ and $\text{ɤ}=\sum_{j=\text{1}}^{n_{i}}\frac{{\left({\text{ɤ}}_{i,j}\right)}^{\text{2}}}{n_{i}}$


$$ℵ=\text{min}\left(\underset{\text{1}\leq j\leq n_{i}}{\text{max}}\left(\left(\left|\text{ɀ}-{\text{ɀ}}_{i,j}\right|+\left|\text{ɤ}-{\text{ɤ}}_{i,j}\right|\right)\right),\text{2}\right).$$

**Proof.** Since $\text{ɀ}=\sum_{j=\text{1}}^{n_{i}}\frac{{\text{ɀ}}_{i,j}}{n_{i}}$ and $\text{ɤ}=\sum_{j=\text{1}}^{n_{i}}\frac{{\text{ɤ}}_{i,j}}{n_{i}}$, then we have


$$\text{ɀ}+\text{ɤ}=\sum_{j=\text{1}}^{n_{i}}\frac{{\left({\text{ɀ}}_{i,j}\right)}^{\text{2}}}{n_{i}}+\sum_{j=\text{1}}^{n_{i}}\frac{{\left({\text{ɤ}}_{i,j}\right)}^{\text{2}}}{n_{i}}=\frac{\sum_{j=\text{1}}^{n_{i}}\left({\left({\text{ɀ}}_{i,j}\right)}^{\text{2}}+{\left({\text{ɤ}}_{i,j}\right)}^{\text{2}}\right)}{n_{i}}\leq \frac{\sum_{j=\text{1}}^{n_{i}}\text{1}}{n_{i}}=\text{1}.$$

Furthermore, it is obvious that ℵ∈[0, 2]. Note that, for Definition 9, we have


$$ℵ=\text{min}\left(\underset{\text{1}\leq j\leq n_{i}}{\text{max}}\left(\left(\left|\text{ɀ}-{\text{ɀ}}_{i,j}\right|+\left|\text{ɤ}-{\text{ɤ}}_{i,j}\right|\right)\right),\text{1}\right).$$

**Example 1.** The following sets of *PyFSs* are represented as:


{(0.3, 0.7), (0.2, 0.7),(0.6, 0.2)},

{(0.2, 0.5), (0.3, 0.4),(0.9, 0.1)},

and


{(0.1, 0.6), (0.5, 0.5),(0.1, 0.8)}.

With the help of Proposition 1, we find the corresponding Dia‑*PyFSs*, we have


(0.37, 0.53;0.63 ), (0.47, 0.33;0.93), (0.23, 0.63;0.53).

### 3.2. Distance measures for Dia‑*PyFSs* via *PyFSs*

The next outcomes are introduced for representing different distances over Dia‑*PyFSs* via Subsection 3 approach.

**Definition 11.** Let *d* be a cardinality of *E*. Then normalized Euclidean distance for two Dia‑*PyFSs* ${\text{Ϣ}}_{{ℵ}_{\text{1}}}^{py}$ and ${\text{Ϣ}}_{{ℵ}_{\text{2}}}^{py}$ is defined as


$$H_{\text{2}}^{q}\left({\text{Ϣ}}_{{ℵ}_{\text{1}}}^{py},\;{\text{Ϣ}}_{{ℵ}_{\text{2}}}^{py}\right)=\frac{\text{1}}{\text{2}}\times \left(\frac{{ℵ}_{\text{1}}-{{ℵ}_{\text{⟠}}}_{\text{2}}}{\text{2}}+\sqrt[{q}]{\frac{\text{1}}{\text{2}d}\sum_{\rho \in E}\left({\left|{\left({\text{ɀ}}_{\text{1}}(\rho )\right)}^{\text{2}}-{\left({\text{ɀ}}_{\text{2}}(\rho )\right)}^{\text{2}}\right|}^{q}+{\left|{\left({\text{ɤ}}_{\text{1}}(\rho )\right)}^{\text{2}}-{\left({\text{ɤ}}_{\text{2}}(\rho )\right)}^{\text{2}}\right|}^{q}\right)}\right),$$

where *q = 1, 2*. If *q = 1* and *q = 2*, then distance $H_{\text{2}}\left({\text{Ϣ}}_{{ℵ}_{\text{1}}}^{py},\;{\text{Ϣ}}_{{ℵ}_{\text{2}}}^{py}\right)$ is known as Hamming distance and Euclidean distance for Dia‑*PyFSs*, respectively.

**Definition 12.** Let *d* be a cardinality of *E*. Then normalized Euclidean distance for two Dia‑*PyFSs* ${\text{Ϣ}}_{{ℵ}_{\text{1}}}^{py}$ and ${\text{Ϣ}}_{{ℵ}_{\text{2}}}^{py}$ is defined as


$$H_{\text{3}}^{q}\left({\text{Ϣ}}_{{ℵ}_{\text{1}}}^{py},\;{\text{Ϣ}}_{{ℵ}_{\text{2}}}^{py}\right)=\frac{\text{1}}{\text{2}}\times \left(\frac{{ℵ}_{\text{1}}-{ℵ}_{\text{2}}}{\text{2}}+\sqrt[{q}]{\frac{\text{1}}{\text{2}d}\sum_{\rho \in E}\left({\left|{\left({\text{ɀ}}_{\text{1}}(\rho )\right)}^{\text{2}}-{\left({\text{ɀ}}_{\text{2}}(\rho )\right)}^{\text{2}}\right|}^{q}+{\left|{\left({\text{ɤ}}_{\text{1}}(\rho )\right)}^{\text{2}}-{\left({\text{ɤ}}_{\text{2}}(\rho )\right)}^{\text{2}}\right|}^{q}+{\left|{\left(\pi_{\text{1}}(\rho )\right)}^{\text{2}}-{\left(\pi_{\text{2}}(\rho )\right)}^{\text{2}}\right|}^{q}\right)}\right),$$

where *q = 1, 2*. If *q = 1* and *q = 2*, then distance $H_{\text{2}}\left({\text{Ϣ}}_{{ℵ}_{\text{1}}}^{py},\;{\text{Ϣ}}_{{ℵ}_{\text{2}}}^{py}\right)$ is known as Szmidt and Kacprzyk’s form of Hamming distance and, Szmidt and Kacprzyk’s form of Euclidean distance for Dia‑*PyFS*s, respectively.

## 4. Diamond Pythagorean fuzzy set related basic operations and relations via max and min approach

The analogs of complement, union, and intersection operations in set theory are defined as follows. Their concepts are inspired by similar definitions within the foundational Dia‑*PyFS* framework.

For the sake of easy understanding, we will take the following three Dia‑*PyFSs* over fixed universe E:


$${\text{Ϣ}}_{{ℵ}_{\text{1}}}^{py}=\left\{\left\langle \rho ,\;{\text{ɀ}}_{\text{1}}(\rho ),\;{\text{ɤ}}_{\text{1}}(\rho );{ℵ}_{\text{1}}\right\rangle :\text{for}\;\text{all}\;\rho \in E\right\},$$

$${\text{Ϣ}}_{{ℵ}_{\text{2}}}^{py}=\left\{\left\langle \rho ,\;{\text{ɀ}}_{\text{2}}(\rho ),\;{\text{ɤ}}_{\text{2}}(\rho );{ℵ}_{\text{2}}\right\rangle :\text{for}\;\text{all}\;\rho \in E\right\},$$

$${\text{Ϣ}}_{{ℵ}_{\text{3}}}^{py}=\left\{\left\langle \rho ,\;{\text{ɀ}}_{\text{3}}(\rho ),\;{\text{ɤ}}_{\text{3}}(\rho );{ℵ}_{\text{3}}\right\rangle :\text{for}\;\text{all}\;\rho \in E\right\}.$$

### 4.1. Operations

Here is some basic operations between two Dia‑*PyFSs* ${\text{Ϣ}}_{{ℵ}_{\text{1}}}^{py}$ and ${\text{Ϣ}}_{{ℵ}_{\text{2}}}^{py}$ are the following:

**Definition 13.** Let ${\text{Ϣ}}_{{ℵ}_{\text{1}}}^{py}$ and ${\text{Ϣ}}_{{ℵ}_{\text{2}}}^{py}$ be two Dia‑*PyFSs*. Then,

i. $\neg {\text{Ϣ}}_{{ℵ}_{\text{1}}}^{py}=\left\{\left\langle \rho ,\;{\text{ɤ}}_{\text{1}}(\rho ),\;{\text{ɀ}}_{\text{1}}(\rho );{ℵ}_{\text{1}}\right\rangle \;:\text{for}\;\text{all}\;\rho \in E\right\}$,
ii. ${\text{Ϣ}}_{{ℵ}_{\text{1}}}^{py}\cup_{\text{𝓂}\text{𝒾}\text{𝓃}}{\text{Ϣ}}_{{ℵ}_{\text{2}}}^{py}=\left\{\left\langle \rho ,\;\text{𝓂}\text{𝒶}\text{𝓍}\left({\text{ɀ}}_{\text{1}}(\rho ),{\text{ɀ}}_{\text{2}}(\rho )\right),\;\text{𝓂}\text{𝒾}\text{𝓃}\left({\text{ɤ}}_{\text{1}}(\rho ),{\text{ɤ}}_{\text{2}}(\rho )\right);\text{𝓂}\text{𝒾}\text{𝓃}\left({ℵ}_{\text{1}},{ℵ}_{\text{2}}\right)\right\rangle \;:\text{for}\;\text{all}\;\rho \in E\right\}$,
iii. ${\text{Ϣ}}_{{ℵ}_{\text{1}}}^{py}\cup_{\text{𝓂}\text{𝒶}\text{𝓍}}{\text{Ϣ}}_{{ℵ}_{\text{2}}}^{py}=\left\{\left\langle \rho ,\;\text{𝓂}\text{𝒶}\text{𝓍}\left({\text{ɀ}}_{\text{1}}(\rho ),{\text{ɀ}}_{\text{2}}(\rho )\right),\;\text{𝓂}\text{𝒾}\text{𝓃}\left({\text{ɤ}}_{\text{1}}(\rho ),{\text{ɤ}}_{\text{2}}(\rho )\right);\text{𝓂}\text{𝒶}\text{𝓍}\left({ℵ}_{\text{1}},{ℵ}_{\text{2}}\right)\right\rangle \;:\text{for}\;\text{all}\;\rho \in E\right\}$,
iv. ${\text{Ϣ}}_{{ℵ}_{\text{1}}}^{py}\cap_{\text{𝓂}\text{𝒾}\text{𝓃}}{\text{Ϣ}}_{{ℵ}_{\text{2}}}^{py}=\left\{\left\langle \rho ,\;\text{𝓂}\text{𝒾}\text{𝓃}\left({\text{ɀ}}_{\text{1}}(\rho ),{\text{ɀ}}_{\text{2}}(\rho )\right),\;\text{𝓂}\text{𝒶}\text{𝓍}\left({\text{ɤ}}_{\text{1}}(\rho ),{\text{ɤ}}_{\text{2}}(\rho )\right);\text{𝓂}\text{𝒾}\text{𝓃}\left({ℵ}_{\text{1}},{ℵ}_{\text{2}}\right)\right\rangle \;:\text{for}\;\text{all}\;\rho \in E\right\}$,
v. ${\text{Ϣ}}_{{ℵ}_{\text{1}}}^{py}\cap_{\text{𝓂}\text{𝒶}\text{𝓍}}{\text{Ϣ}}_{{ℵ}_{\text{2}}}^{py}=\left\{\left\langle \rho ,\;\text{𝓂}\text{𝒾}\text{𝓃}\left({\text{ɀ}}_{\text{1}}(\rho ),{\text{ɀ}}_{\text{2}}(\rho )\right),\;\text{𝓂}\text{𝒶}\text{𝓍}\left({\text{ɤ}}_{\text{1}}(\rho ),{\text{ɤ}}_{\text{2}}(\rho )\right);\text{𝓂}\text{𝒶}\text{𝓍}\left({ℵ}_{\text{1}},{ℵ}_{\text{2}}\right)\right\rangle \;:\text{for}\;\text{all}\;\rho \in E\right\}$,
vi. ${\text{Ϣ}}_{{ℵ}_{\text{1}}}^{py}{⨂}_{\text{𝓂}\text{𝒾}\text{𝓃}}{\text{Ϣ}}_{{ℵ}_{\text{2}}}^{py}=\left({\text{ɀ}}_{\text{1}}(\rho )\cdot {\text{ɀ}}_{\text{2}}(\rho ),\;\sqrt{{\left({\text{ɤ}}_{\text{1}}(\rho )\right)}^{\text{2}}+{\left({\text{ɤ}}_{\text{2}}(\rho )\right)}^{\text{2}}-{\left({\text{ɤ}}_{\text{1}}(\rho )\right)}^{\text{2}}\cdot {\left({\text{ɤ}}_{\text{2}}(\rho )\right)}^{\text{2}}}\text{𝓂}\text{𝒾}\text{𝓃}\left({ℵ}_{\text{1}},{ℵ}_{\text{2}}\right)\right)$,
vii. ${\text{Ϣ}}_{{ℵ}_{\text{1}}}^{py}{⨂}_{\text{𝓂}\text{𝒶}\text{𝓍}}{\text{Ϣ}}_{{ℵ}_{\text{2}}}^{py}=\left({\text{ɀ}}_{\text{1}}(\rho )\cdot {\text{ɀ}}_{\text{2}}(\rho ),\;\sqrt{{\left({\text{ɤ}}_{\text{1}}(\rho )\right)}^{\text{2}}+{\left({\text{ɤ}}_{\text{2}}(\rho )\right)}^{\text{2}}-{\left({\text{ɤ}}_{\text{1}}(\rho )\right)}^{\text{2}}\cdot {\left({\text{ɤ}}_{\text{2}}(\rho )\right)}^{\text{2}}}\text{𝓂}\text{𝒶}\text{𝓍}\left({ℵ}_{\text{1}},{ℵ}_{\text{2}}\right)\right)$,
viii. ${\text{Ϣ}}_{{ℵ}_{\text{1}}}^{py}{⨁}_{\text{𝓂}\text{𝒾}\text{𝓃}}{\text{Ϣ}}_{{ℵ}_{\text{2}}}^{py}=\left(\sqrt{{\left({\text{ɀ}}_{\text{1}}(\rho )\right)}^{\text{2}}+{\left({\text{ɀ}}_{\text{2}}(\rho )\right)}^{\text{2}}-{\left({\text{ɀ}}_{\text{1}}(\rho )\right)}^{\text{2}}\cdot {\left({\text{ɀ}}_{\text{2}}(\rho )\right)}^{\text{2}}},\;{\text{ɤ}}_{\text{1}}(\rho )\cdot {\text{ɤ}}_{\text{2}}(\rho text𝓂\text{𝒾}\text{𝓃}\left({ℵ}_{\text{1}},{ℵ}_{\text{2}}\right)\right)$,
ix. ${\text{Ϣ}}_{{ℵ}_{\text{1}}}^{py}{⨁}_{\text{𝓂}\text{𝒶}\text{𝓍}}{\text{Ϣ}}_{{ℵ}_{\text{2}}}^{py}=\left(\sqrt{{\left({\text{ɀ}}_{\text{1}}(\rho )\right)}^{\text{2}}+{\left({\text{ɀ}}_{\text{2}}(\rho )\right)}^{\text{2}}-{\left({\text{ɀ}}_{\text{1}}(\rho )\right)}^{\text{2}}\cdot {\left({\text{ɀ}}_{\text{2}}(\rho )\right)}^{\text{2}}},\;{\text{ɤ}}_{\text{1}}(\rho )\cdot {\text{ɤ}}_{\text{2}}(\rho text𝓂\text{𝒶}\text{𝓍}\left({ℵ}_{\text{1}},{ℵ}_{\text{2}}\right)\right)\;,$
x. $\text{ƛ}{\text{Ϣ}}_{{ℵ}_{\text{1}}}^{py}=\left(\sqrt{\text{1}-{\left(\text{1}-{\left({\text{ɀ}}_{\text{1}}(\rho )\right)}^{\text{2}}\right)}^{\text{ƛ}}},\;{{\text{ɤ}}_{\text{1}}(\rho )}^{\text{ƛ}};{ℵ}_{\text{1}}\right)$; ƛ>0,
xi. ${{\text{Ϣ}}_{{ℵ}_{\text{1}}}^{py}}^{\text{ƛ}}=\left({{\text{ɀ}}_{\text{1}}(\rho )}^{\text{ƛ}},\;\sqrt{\text{1}-{\left(\text{1}-{\left({\text{ɤ}}_{\text{1}}(\rho )\right)}^{\text{2}}\right)}^{\text{ƛ}}};{ℵ}_{\text{1}}\right),$

### 4.2. Relations

The relations over Dia‑*PyFSs* are firstly proposed as follows:

**Definition 14.** Let ${\text{Ϣ}}_{{ℵ}_{\text{1}}}^{py}$ and ${{\text{Ϣ}}_{ℵ}^{py}}_{\text{2}}$ be two Dia‑*PyFSs*. Then, for all ρ∈E, we have

- ${\text{Ϣ}}_{{ℵ}_{\text{1}}}^{py}\subset {\text{Ϣ}}_{{ℵ}_{\text{2}}}^{py}$ iff $\left(({ℵ}_{\text{1}}<{ℵ}_{\text{2}}\&\left(\begin{array}{c} @r@\left({\text{ɀ}}_{\text{1}}(\rho )<{\text{ɀ}}_{\text{2}}(\rho )\&\;{\text{ɤ}}_{\text{1}}(\rho )\geq {\text{ɤ}}_{\text{2}}(\rho )\right)\\ \vee \left({\text{ɀ}}_{\text{1}}(\rho )\leq {\text{ɀ}}_{\text{2}}(\rho )\&\;{\text{ɤ}}_{\text{1}}(\rho )>{\text{ɤ}}_{\text{2}}(\rho )\right)\\ \vee \left({\text{ɀ}}_{\text{1}}(\rho )<{\text{ɀ}}_{\text{2}}(\rho )\&\;{\text{ɤ}}_{\text{1}}(\rho )>{\text{ɤ}}_{\text{2}}(\rho )\right) \end{array}\;\right)\;\right)\;;$
- ${\text{Ϣ}}_{{ℵ}_{\text{1}}}^{py}\subset {\text{Ϣ}}_{{ℵ}_{\text{2}}}^{py}$ iff ${\text{Ϣ}}_{{ℵ}_{\text{2}}}^{py}⊃{{\text{Ϣ}}_{ℵ}^{py}}_{\text{1}}$
- ${\text{Ϣ}}_{{ℵ}_{\text{1}}}^{py}\subseteq {\text{Ϣ}}_{{ℵ}_{\text{2}}}^{py}$ iff $\left(({ℵ}_{\text{1}}\leq {ℵ}_{\text{2}}\&{\;\text{ɀ}}_{\text{1}}(\rho )\leq {\text{ɀ}}_{\text{2}}(\rho )\;\&{\;\text{ɤ}}_{\text{1}}(\rho )\geq {\text{ɤ}}_{\text{2}}(\rho )\right);$
- ${\text{Ϣ}}_{{ℵ}_{\text{1}}}^{py}\subseteq {\text{Ϣ}}_{{ℵ}_{\text{2}}}^{py}$ iff ${\text{Ϣ}}_{{ℵ}_{\text{2}}}^{py}⊇{\text{Ϣ}}_{{ℵ}_{\text{1}}}^{py}$
- ${\text{Ϣ}}_{{ℵ}_{\text{1}}}^{py}={\text{Ϣ}}_{{ℵ}_{\text{2}}}^{py}$ iff $({ℵ}_{\text{1}}={ℵ}_{\text{2}}\&{\;\text{ɀ}}_{\text{1}}(\rho )={\text{ɀ}}_{\text{2}}(\rho )\;\&{\;\text{ɤ}}_{\text{1}}(\rho )={\text{ɤ}}_{\text{2}}(\rho )$.

From Definitions 13 and 14, we have concluded the following results:

**Proposition 2.** Let ${\text{Ϣ}}_{{ℵ}_{\text{1}}}^{py}$, and ${\text{Ϣ}}_{{ℵ}_{\text{2}}}^{py}$ be two Dia‑*PyFSs*. Then, following properties holds such that

- $\neg \left(\neg {\text{Ϣ}}_{{ℵ}_{\text{1}}}^{py}\cup_{\text{𝓂}\text{𝒾}\text{𝓃}}\neg {\text{Ϣ}}_{{ℵ}_{\text{2}}}^{py}\right)={\text{Ϣ}}_{{ℵ}_{\text{1}}}^{py}\cap_{\text{𝓂}\text{𝒾}\text{𝓃}}{\text{Ϣ}}_{{ℵ}_{\text{2}}}^{py}$, and $\neg \left(\neg {\text{Ϣ}}_{{ℵ}_{\text{1}}}^{py}\cup_{\text{𝓂}\text{𝒶}\text{𝓍}}\neg {\text{Ϣ}}_{{ℵ}_{\text{2}}}^{py}\right)={\text{Ϣ}}_{{ℵ}_{\text{1}}}^{py}\cap_{\text{𝓂}\text{𝒶}\text{𝓍}}{\text{Ϣ}}_{{ℵ}_{\text{2}}}^{py}$,
- $\neg \left(\neg {\text{Ϣ}}_{{ℵ}_{\text{1}}}^{py}\cap_{\text{𝓂}\text{𝒾}\text{𝓃}}\neg {\text{Ϣ}}_{{ℵ}_{\text{2}}}^{py}\right)={\text{Ϣ}}_{{ℵ}_{\text{1}}}^{py}\cup_{\text{𝓂}\text{𝒾}\text{𝓃}}{\text{Ϣ}}_{{ℵ}_{\text{2}}}^{py}$, and $\neg \left(\neg {\text{Ϣ}}_{{ℵ}_{\text{1}}}^{py}\cap_{\text{𝓂}\text{𝒶}\text{𝓍}}\neg {\text{Ϣ}}_{{ℵ}_{\text{2}}}^{py}\right)={\text{Ϣ}}_{{ℵ}_{\text{1}}}^{py}\cup_{\text{𝓂}\text{𝒶}\text{𝓍}}{\text{Ϣ}}_{{ℵ}_{\text{2}}}^{py}$,

**Proof.** The proof follows similar steps as those used in the operations of *PyFSs*, and as such, is excluded for brevity.

**Proposition 3.** Let ${{\text{Ϣ}}_{ℵ}^{py}}_{\text{1}}$, ${\text{Ϣ}}_{{ℵ}_{\text{2}}}^{py}$ and ${\text{Ϣ}}_{{ℵ}_{\text{3}}}^{py}$ be three Dia‑*PyFSs*. Then, following properties holds such that

1) ${\text{Ϣ}}_{{ℵ}_{\text{1}}}^{py}\cup_{\text{𝓂}\text{𝒾}\text{𝓃}}{\text{Ϣ}}_{{ℵ}_{\text{1}}}^{py}={\text{Ϣ}}_{{ℵ}_{\text{1}}}^{py}$,and ${\text{Ϣ}}_{{ℵ}_{\text{1}}}^{py}\cup_{\text{𝓂}\text{𝒶}\text{𝓍}}{\text{Ϣ}}_{{ℵ}_{\text{1}}}^{py}={\text{Ϣ}}_{{ℵ}_{\text{1}}}^{py}$,
2) ${\text{Ϣ}}_{{ℵ}_{\text{1}}}^{py}\cap_{\text{𝓂}\text{𝒾}\text{𝓃}}{\text{Ϣ}}_{{ℵ}_{\text{1}}}^{py}={{\text{Ϣ}}_{ℵ}^{py}}_{\text{1}}$, and ${\text{Ϣ}}_{{ℵ}_{\text{1}}}^{py}\cap_{\text{𝓂}\text{𝒶}\text{𝓍}}{\text{Ϣ}}_{{ℵ}_{\text{1}}}^{py}={\text{Ϣ}}_{{ℵ}_{\text{1}}}^{py}$,
3) ${\text{Ϣ}}_{{ℵ}_{\text{1}}}^{py}\cup_{\text{𝓂}\text{𝒾}\text{𝓃}}{\text{Ϣ}}_{{ℵ}_{\text{2}}}^{py}={\text{Ϣ}}_{{ℵ}_{\text{2}}}^{py}\cup_{\text{𝓂}\text{𝒾}\text{𝓃}}{\text{Ϣ}}_{{ℵ}_{\text{1}}}^{py}$,and ${\text{Ϣ}}_{{ℵ}_{\text{1}}}^{py}\cup_{\text{𝓂}\text{𝒶}\text{𝓍}}{\text{Ϣ}}_{{ℵ}_{\text{2}}}^{py}={\text{Ϣ}}_{{ℵ}_{\text{2}}}^{py}\cup_{\text{𝓂}\text{𝒶}\text{𝓍}}{\text{Ϣ}}_{{ℵ}_{\text{1}}}^{py}$,
4) ${\text{Ϣ}}_{{ℵ}_{\text{1}}}^{py}\cap_{\text{𝓂}\text{𝒾}\text{𝓃}}{\text{Ϣ}}_{{ℵ}_{\text{2}}}^{py}={\text{Ϣ}}_{{ℵ}_{\text{2}}}^{py}\cap_{\text{𝓂}\text{𝒾}\text{𝓃}}{\text{Ϣ}}_{{ℵ}_{\text{1}}}^{py}$, and ${\text{Ϣ}}_{{ℵ}_{\text{1}}}^{py}\cap_{\text{𝓂}\text{𝒶}\text{𝓍}}{\text{Ϣ}}_{{ℵ}_{\text{2}}}^{py}={\text{Ϣ}}_{{ℵ}_{\text{2}}}^{py}\cap_{\text{𝓂}\text{𝒶}\text{𝓍}}{\text{Ϣ}}_{{ℵ}_{\text{1}}}^{py}$,
5) ${\text{Ϣ}}_{{ℵ}_{\text{1}}}^{py}\cup_{\text{𝓂}\text{𝒾}\text{𝓃}}\left({\text{Ϣ}}_{{ℵ}_{\text{2}}}^{py}\cup_{\text{𝓂}\text{𝒾}\text{𝓃}}{\text{Ϣ}}_{{ℵ}_{\text{3}}}^{py}\right)=\left({\text{Ϣ}}_{{ℵ}_{\text{1}}}^{py}\cup_{\text{𝓂}\text{𝒾}\text{𝓃}}{{\text{Ϣ}}_{ℵ}^{py}}_{\text{1}}\right)\cup_{\text{𝓂}\text{𝒾}\text{𝓃}}{\text{Ϣ}}_{{ℵ}_{\text{3}}}^{py}$ and ${\text{Ϣ}}_{{ℵ}_{\text{1}}}^{py}\cup_{\text{𝓂}\text{𝒶}\text{𝓍}}\left({\text{Ϣ}}_{{ℵ}_{\text{2}}}^{py}\cup_{\text{𝓂}\text{𝒶}\text{𝓍}}{\text{Ϣ}}_{{ℵ}_{\text{3}}}^{py}\right)=\left({\text{Ϣ}}_{{ℵ}_{\text{1}}}^{py}\cup_{\text{𝓂}\text{𝒶}\text{𝓍}}{\text{Ϣ}}_{{ℵ}_{\text{2}}}^{py}\right)\cup_{\text{𝓂}\text{𝒶}\text{𝓍}}{\text{Ϣ}}_{{ℵ}_{\text{3}}}^{py}$,
6) ${\text{Ϣ}}_{{ℵ}_{\text{1}}}^{py}\cap_{\text{𝓂}\text{𝒾}\text{𝓃}}\left({\text{Ϣ}}_{{ℵ}_{\text{2}}}^{py}\cap_{\text{𝓂}\text{𝒾}\text{𝓃}}{\text{Ϣ}}_{{ℵ}_{\text{3}}}^{py}\right)=\left({\text{Ϣ}}_{{ℵ}_{\text{1}}}^{py}\cap_{\text{𝓂}\text{𝒾}\text{𝓃}}{\text{Ϣ}}_{{ℵ}_{\text{2}}}^{py}\right)\cap_{\text{𝓂}\text{𝒾}\text{𝓃}}{\text{Ϣ}}_{{ℵ}_{\text{3}}}^{py}$ and ${\text{Ϣ}}_{{ℵ}_{\text{1}}}^{py}\cap_{\text{𝓂}\text{𝒶}\text{𝓍}}\left({\text{Ϣ}}_{{ℵ}_{\text{2}}}^{py}\cap_{\text{𝓂}\text{𝒶}\text{𝓍}}{\text{Ϣ}}_{{ℵ}_{\text{3}}}^{py}\right)=\left({\text{Ϣ}}_{{ℵ}_{\text{1}}}^{py}\cap_{\text{𝓂}\text{𝒶}\text{𝓍}}{\text{Ϣ}}_{{ℵ}_{\text{2}}}^{py}\right)\cap_{\text{𝓂}\text{𝒶}\text{𝓍}}{\text{Ϣ}}_{{ℵ}_{\text{3}}}^{py}$,
7) ${\text{Ϣ}}_{{ℵ}_{\text{1}}}^{py}\cap_{\text{𝓂}\text{𝒾}\text{𝓃}}\left({\text{Ϣ}}_{{ℵ}_{\text{2}}}^{py}\cup_{\text{𝓂}\text{𝒾}\text{𝓃}}{\text{Ϣ}}_{{ℵ}_{\text{3}}}^{py}\right)=\left({\text{Ϣ}}_{{ℵ}_{\text{1}}}^{py}\cap_{\text{𝓂}\text{𝒾}\text{𝓃}}{\text{Ϣ}}_{{ℵ}_{\text{2}}}^{py}\right)\cup_{\text{𝓂}\text{𝒾}\text{𝓃}}\left({\text{Ϣ}}_{{ℵ}_{\text{1}}}^{py}\cap_{\text{𝓂}\text{𝒾}\text{𝓃}}{\text{Ϣ}}_{{ℵ}_{\text{3}}}^{py}\right)$ and ${\text{Ϣ}}_{{ℵ}_{\text{1}}}^{py}\cap_{\text{𝓂}\text{𝒶}\text{𝓍}}\left({\text{Ϣ}}_{{ℵ}_{\text{2}}}^{py}\cup_{\text{𝓂}\text{𝒾}\text{𝓃}}{\text{Ϣ}}_{{ℵ}_{\text{3}}}^{py}\right)=\left({\text{Ϣ}}_{{ℵ}_{\text{1}}}^{py}\cap_{\text{𝓂}\text{𝒶}\text{𝓍}}{\text{Ϣ}}_{{ℵ}_{\text{2}}}^{py}\right)\cup_{\text{𝓂}\text{𝒾}\text{𝓃}}\left({\text{Ϣ}}_{{ℵ}_{\text{1}}}^{py}\cap_{\text{𝓂}\text{𝒶}\text{𝓍}}{\text{Ϣ}}_{{ℵ}_{\text{3}}}^{py}\right)$,
8) ${\text{Ϣ}}_{{ℵ}_{\text{1}}}^{py}\cap_{\text{𝓂}\text{𝒾}\text{𝓃}}\left({\text{Ϣ}}_{{ℵ}_{\text{2}}}^{py}\cup_{\text{𝓂}\text{𝒶}\text{𝓍}}{\text{Ϣ}}_{{ℵ}_{\text{3}}}^{py}\right)=\left({\text{Ϣ}}_{{ℵ}_{\text{1}}}^{py}\cap_{\text{𝓂}\text{𝒾}\text{𝓃}}{\text{Ϣ}}_{{ℵ}_{\text{2}}}^{py}\right)\cup_{\text{𝓂}\text{𝒶}\text{𝓍}}\left({\text{Ϣ}}_{{ℵ}_{\text{1}}}^{py}\cap_{\text{𝓂}\text{𝒾}\text{𝓃}}{\text{Ϣ}}_{{ℵ}_{\text{3}}}^{py}\right)$ and ${\text{Ϣ}}_{{ℵ}_{\text{1}}}^{py}\cap_{\text{𝓂}\text{𝒶}\text{𝓍}}\left({\text{Ϣ}}_{{ℵ}_{\text{2}}}^{py}\cup_{\text{𝓂}\text{𝒶}\text{𝓍}}{\text{Ϣ}}_{{ℵ}_{\text{3}}}^{py}\right)=\left({\text{Ϣ}}_{{ℵ}_{\text{1}}}^{py}\cap_{\text{𝓂}\text{𝒶}\text{𝓍}}{\text{Ϣ}}_{{ℵ}_{\text{2}}}^{py}\right)\cup_{\text{𝓂}\text{𝒶}\text{𝓍}}\left({\text{Ϣ}}_{{ℵ}_{\text{1}}}^{py}\cap_{\text{𝓂}\text{𝒶}\text{𝓍}}{\text{Ϣ}}_{{ℵ}_{\text{3}}}^{py}\right)$,
9) ${\text{Ϣ}}_{{ℵ}_{\text{1}}}^{py}\cup_{\text{𝓂}\text{𝒾}\text{𝓃}}\left({\text{Ϣ}}_{{ℵ}_{\text{2}}}^{py}\cap_{\text{𝓂}\text{𝒾}\text{𝓃}}{\text{Ϣ}}_{{ℵ}_{\text{3}}}^{py}\right)=\left({\text{Ϣ}}_{{ℵ}_{\text{1}}}^{py}\cup_{\text{𝓂}\text{𝒾}\text{𝓃}}{\text{Ϣ}}_{{ℵ}_{\text{2}}}^{py}\right)\cap_{\text{𝓂}\text{𝒾}\text{𝓃}}\left({\text{Ϣ}}_{{ℵ}_{\text{1}}}^{py}\cup_{\text{𝓂}\text{𝒾}\text{𝓃}}{\text{Ϣ}}_{{ℵ}_{\text{3}}}^{py}\right)$ and ${\text{Ϣ}}_{{ℵ}_{\text{1}}}^{py}\cup_{\text{𝓂}\text{𝒶}\text{𝓍}}\left({\text{Ϣ}}_{{ℵ}_{\text{2}}}^{py}\cap_{\text{𝓂}\text{𝒾}\text{𝓃}}{\text{Ϣ}}_{{ℵ}_{\text{3}}}^{py}\right)=\left({\text{Ϣ}}_{{ℵ}_{\text{1}}}^{py}\cup_{\text{𝓂}\text{𝒶}\text{𝓍}}{\text{Ϣ}}_{{ℵ}_{\text{2}}}^{py}\right)\cap_{\text{𝓂}\text{𝒾}\text{𝓃}}\left({\text{Ϣ}}_{{ℵ}_{\text{1}}}^{py}\cup_{\text{𝓂}\text{𝒶}\text{𝓍}}{\text{Ϣ}}_{{ℵ}_{\text{3}}}^{py}\right)$,
10) ${\text{Ϣ}}_{{ℵ}_{\text{1}}}^{py}\cup_{\text{𝓂}\text{𝒾}\text{𝓃}}\left({\text{Ϣ}}_{{ℵ}_{\text{2}}}^{py}\cap_{\text{𝓂}\text{𝒶}\text{𝓍}}{\text{Ϣ}}_{{ℵ}_{\text{3}}}^{py}\right)=\left({\text{Ϣ}}_{{ℵ}_{\text{1}}}^{py}\cup_{\text{𝓂}\text{𝒾}\text{𝓃}}{\text{Ϣ}}_{{ℵ}_{\text{2}}}^{py}\right)\cap_{\text{𝓂}\text{𝒶}\text{𝓍}}\left({\text{Ϣ}}_{{ℵ}_{\text{1}}}^{py}\cup_{\text{𝓂}\text{𝒾}\text{𝓃}}{\text{Ϣ}}_{{ℵ}_{\text{3}}}^{py}\right)$ and ${\text{Ϣ}}_{{ℵ}_{\text{1}}}^{py}\cup_{\text{𝓂}\text{𝒶}\text{𝓍}}\left({\text{Ϣ}}_{{ℵ}_{\text{2}}}^{py}\cap_{\text{𝓂}\text{𝒶}\text{𝓍}}{\text{Ϣ}}_{{ℵ}_{\text{3}}}^{py}\right)=\left({\text{Ϣ}}_{{ℵ}_{\text{1}}}^{py}\cup_{\text{𝓂}\text{𝒶}\text{𝓍}}{\text{Ϣ}}_{{ℵ}_{\text{2}}}^{py}\right)\cap_{\text{𝓂}\text{𝒶}\text{𝓍}}\left({\text{Ϣ}}_{{ℵ}_{\text{1}}}^{py}\cup_{\text{𝓂}\text{𝒶}\text{𝓍}}{\text{Ϣ}}_{{ℵ}_{\text{3}}}^{py}\right).$

**Proof.** The proof follows a process similar to that used in the operations of *PyFSs* and is therefore omitted for the sake of brevity.

To keep things concise, it is noted that for the upcoming results, ℵ∈[0,1].

## 5. Diamond Pythagorean fuzzy set related operations

The following definitions pertain to arithmetic operations for Dia‑*PyFSs* via *𝓉*-norm and *𝓉*-conorm, including addition, multiplication, and exponentiation:

**Definition 15.** Let ${\text{Ϣ}}_{{ℵ}_{\text{1}}}^{py}=\left\langle {\text{ɀ}}_{\text{1}},\;{\text{ɤ}}_{\text{1}};{ℵ}_{\text{1}}\right\rangle$ and ${\text{Ϣ}}_{{ℵ}_{\text{2}}}^{py}=\left\langle {\text{ɀ}}_{\text{2}},\;{\text{ɤ}}_{\text{2}};{ℵ}_{\text{2}}\right\rangle$ be two Dia‑*PyFSs*. Assume that, in the context of diamond Pythagorean fuzzy complement, $N(\hbar )={\left(\text{1}-\hbar^{\text{2}}\right)}^{\frac{\text{1}}{\text{2}}}$ with ϙ as the norm or conorm, T and S as the dual *𝓉*-norm and *𝓉*-conorm, respectively. The general algebraic operations among Dia‑*PyFSs* are defined as follows:

1) T,
2) T.

It is clear that the operations presented in Definition 15 are based on those outlined in Definition 13, with particular selections for T, S, and ϙ.

**Proposition 4.** Let ${\text{Ϣ}}_{{ℵ}_{\text{1}}}^{py}=\left\langle {\text{ɀ}}_{\text{1}},\;{\text{ɤ}}_{\text{1}};{ℵ}_{\text{1}}\right\rangle$ and ${\text{Ϣ}}_{{ℵ}_{\text{1}}}^{py}=\left\langle {\text{ɀ}}_{\text{2}},\;{\text{ɤ}}_{\text{2}};{ℵ}_{\text{2}}\right\rangle$ be two Dia‑*PyFSs*. Assume that, in the context of Pythagorean fuzzy complement, $N(\hbar )={\left(\text{1}-\hbar^{\text{2}}\right)}^{\frac{\text{1}}{\text{2}}}$ with ϙ as the norm or conorm, T and S as the dual *𝓉*-norm and *𝓉*-conorm, respectively. Then ${\text{Ϣ}}_{{ℵ}_{\text{1}}}^{py}{⨁}_{\text{ϙ}}{\text{Ϣ}}_{{ℵ}_{\text{2}}}^{py}$ and ${\text{Ϣ}}_{{ℵ}_{\text{1}}}^{py}{⨂}_{\text{ϙ}}{\text{Ϣ}}_{{ℵ}_{\text{2}}}^{py}$ are also Dia‑*PyFS*.

Proof. Sine *S* is a dual *𝓉*-conorm corresponding to Pythagorean fuzzy complement *N*, then $S\left({\text{ɤ}}_{\text{1}},{\text{ɤ}}_{\text{2}}\right)={\left(\text{1}-T^{\text{2}}\left({\left(\text{1}-{{\text{ɤ}}_{\text{1}}}^{\text{2}}\right)}^{\frac{\text{1}}{\text{2}}},{\left(\text{1}-{{\text{ɤ}}_{\text{2}}}^{\text{2}}\right)}^{\frac{\text{1}}{\text{2}}}\right)\right)}^{\frac{\text{1}}{\text{2}}}$. We know that $\text{ɀ}\leq {\left(\text{1}-{\text{ɤ}}^{\text{2}}\right)}^{\frac{\text{1}}{\text{2}}}$ and T is nondecreasing, then we have


$$\begin{array}{cc} T^{\text{2}}\left({\text{ɀ}}_{\text{1}},{\text{ɀ}}_{\text{2}}\right)+S^{\text{2}}\left({\text{ɤ}}_{\text{1}},{\text{ɤ}}_{\text{2}}\right) & =T^{\text{2}}\left({\text{ɀ}}_{\text{1}},{\text{ɀ}}_{\text{2}}\right)+{\left({\left(\text{1}-T^{\text{2}}\left({\left(\text{1}-{{\text{ɤ}}_{\text{1}}}^{\text{2}}\right)}^{\frac{\text{1}}{\text{2}}},{\left(\text{1}-{{\text{ɤ}}_{\text{2}}}^{\text{2}}\right)}^{\frac{\text{1}}{\text{2}}}\right)\right)}^{\frac{\text{1}}{\text{2}}}\right)}^{\text{2}}\\ & \leq T^{\text{2}}\left({\left(\text{1}-{{\text{ɤ}}_{\text{1}}}^{\text{2}}\right)}^{\frac{\text{1}}{\text{2}}},{\left(\text{1}-{{\text{ɤ}}_{\text{2}}}^{\text{2}}\right)}^{\frac{\text{1}}{\text{2}}}\right)+\text{1}-T^{\text{2}}\left({\left(\text{1}-{{\text{ɤ}}_{\text{1}}}^{\text{2}}\right)}^{\frac{\text{1}}{\text{2}}},{\left(\text{1}-{{\text{ɤ}}_{\text{2}}}^{\text{2}}\right)}^{\frac{\text{1}}{\text{2}}}\right)=\text{1}. \end{array}$$

Moreover, since the domain of *ϙ* is the closed unit interval, we conclude that ${\text{Ϣ}}_{{ℵ}_{\text{1}}}^{py}{⨂}_{\text{ϙ}}{\text{Ϣ}}_{{ℵ}_{\text{2}}}^{py}$ is a Dia‑*PyFS*. It can also be shown that ${\text{Ϣ}}_{{ℵ}_{\text{1}}}^{py}{⨁}_{\text{ϙ}}{\text{Ϣ}}_{{ℵ}_{\text{2}}}^{py}$ is a Dia‑*PyFV*.

As a result, algebraic operations on Dia‑*PyFV*s can be defined using the additive generators of strict Archimedean *𝓉*-norms and *𝓉*-conorms.

**Definition 16.** Let ƛ>0, and assume that $\text{Ϣ}=\left\langle {\text{ɀ}}_{\text{Ϣ}},{\text{ɤ}}_{\text{Ϣ}};{ℵ}_{\text{Ϣ}}\right\rangle$ and $L=\left\langle {\text{ɀ}}_{L},{\text{ɤ}}_{L};{ℵ}_{L}\right\rangle$ are two Dia‑*PyFV*s. Suppose that the additive generator of a continuous Archimedean *𝓉*-norm is 𝒷:[0,1]→[0,∞], and the additive generator of a continuous Archimedean *𝓉*-norm or *𝓉*-conorm is ϙ:[0,1]→[0,∞], with $h(\text{𝓉})=\text{𝒷}\left({\left(\text{1}-{\text{𝓉}}^{\text{2}}\right)}^{\frac{\text{1}}{\text{2}}}\right)$. The following definitions describe algebraic operations for Dia‑*PyFV*:

i. $\text{Ϣ}{⊕}_{\text{ϙ}}L=\left\langle h^{-\text{1}}\left(h\left({\text{ɀ}}_{\text{Ϣ}}\right)+h\left({\text{ɀ}}_{L}\right)\right),{\text{𝒷}}^{-\text{1}}\left(\text{𝒷}\left({\text{ɤ}}_{\text{Ϣ}}\right)+\text{𝒷}\left({\text{ɤ}}_{L}\right)\right);{\text{ϙ}}^{-\text{1}}\left(\text{ϙ}\left({ℵ}_{\text{Ϣ}}\right)+\text{ϙ}\left({ℵ}_{L}\right)\right)\right\rangle ,$
ii. $\text{Ϣ}{⊗}_{\text{ϙ}}L=\left\langle {\text{𝒷}}^{-\text{1}}\left(\text{𝒷}\left({\text{ɀ}}_{\text{Ϣ}}\right)+\text{𝒷}\left({\text{ɀ}}_{L}\right)\right),h^{-\text{1}}\left(h\left({\text{ɤ}}_{\text{Ϣ}}\right)+h\left({\text{ɤ}}_{L}\right)\right);{\text{ϙ}}^{-\text{1}}\left(\text{ϙ}\left({ℵ}_{\text{Ϣ}}\right)+\text{ϙ}\left({ℵ}_{L}\right)\right)\right\rangle ,$
iii. ${\text{ƛ}}_{\text{ϙ}}\text{Ϣ}=\left\langle h^{-\text{1}}\left(\text{ƛ}h\left({\text{ɀ}}_{\text{Ϣ}}\right)\right),{\text{𝒷}}^{-\text{1}}\left(\text{ƛ}b\left({\text{ɤ}}_{\text{Ϣ}}\right)\right);{\text{ϙ}}^{-\text{1}}\left(\text{ƛ}\text{ϙ}\left({ℵ}_{\text{Ϣ}}\right)\right)\right\rangle ,$
iv. ${\text{Ϣ}}^{{\text{ƛ}}_{\text{ϙ}}}=\left\langle {\text{𝒷}}^{-\text{1}}\left(\text{ƛ}b\left({\text{ɀ}}_{\text{Ϣ}}\right)\right),h^{-\text{1}}\left(\text{ƛ}h\left({\text{ɤ}}_{\text{Ϣ}}\right)\right);{\text{ϙ}}^{-\text{1}}\left(\text{ƛ}\text{ϙ}\left({ℵ}_{\text{Ϣ}}\right)\right)\right\rangle .$

The following statement verifies that Dia‑*PyFV*s are also multiplication by constant and power of Dia‑*PyFV*s.

**Proposition 5.** Let ƛ>0, and assume that $\text{Ϣ}=\left\langle {\text{ɀ}}_{\text{Ϣ}},{\text{ɤ}}_{\text{Ϣ}};{ℵ}_{\text{Ϣ}}\right\rangle$ and $L=\left\langle {\text{ɀ}}_{L},{\text{ɤ}}_{L};{ℵ}_{L}\right\rangle$ are two Dia‑*PyFV*s. Suppose that the additive generator of a continuous Archimedean *𝓉*-norm is 𝒷:[0,1]→[0,∞], and the additive generator of a continuous Archimedean *𝓉*-norm or *𝓉*-conorm is ϙ:[0,1]→[0,∞], with $h(\text{𝓉})=\text{𝒷}\left({\left(\text{1}-{\text{𝓉}}^{\text{2}}\right)}^{\frac{\text{1}}{\text{2}}}\right)$. Then $\text{Ϣ}{⊕}_{\text{ϙ}}L$, $\text{Ϣ}{⊗}_{\text{ϙ}}L$, ${\text{ƛ}}_{\text{ϙ}}\text{Ϣ}$ and ${\text{Ϣ}}^{{\text{ƛ}}_{\text{ϙ}}}$.

**Proof.** The Proposition 5 makes it obvious that $\text{Ϣ}{⊕}_{\text{ϙ}}L$ and $\text{Ϣ}{⊗}_{\text{ϙ}}L$ are ${\text{Ϣ}}^{py}\text{-}PyFV\text{s}$. It is well known that $h^{-\text{1}}\left(\text{𝓉}\right)={\left(\text{1}-{\left[{\text{𝒷}}^{-\text{1}}\left(\text{𝓉}\right)\right]}^{\text{2}}\right)}^{\frac{\text{1}}{\text{2}}}$ and $\text{𝒷}(\text{𝓉})=h\left({\left(\text{1}-{\text{𝓉}}^{\text{2}}\right)}^{\frac{\text{1}}{\text{2}}}\right)$. Now, $\text{ɀ}\leq {\left(\text{1}-{\text{ɤ}}^{\text{2}}\right)}^{\frac{\text{1}}{\text{2}}}$ and $h,h^{-\text{1}}$ are non-decreasing, then


$$\begin{array}{cc} \text{0} & \leq {\left(h^{-\text{1}}\left(\text{ƛ}h\left({\text{ɀ}}_{\text{Ϣ}}\right)\right)\right)}^{\text{2}}+{\left({\text{𝒷}}^{-\text{1}}\left(\text{ƛ}b\left({\text{ɤ}}_{\text{Ϣ}}\right)\right)\right)}^{\text{2}}\leq {\left(h^{-\text{1}}\left(\text{ƛ}h\left(\text{1}-{\text{ɤ}}_{\text{Ϣ}}\right)\right)\right)}^{\text{2}}+{\left({\text{𝒷}}^{-\text{1}}\left(\text{ƛ}b\left({\text{ɤ}}_{\text{Ϣ}}\right)\right)\right)}^{\text{2}}\\ & =\text{1}-{\left({\text{𝒷}}^{-\text{1}}\left(\text{ƛ}h\left(\text{1}-{\text{ɤ}}_{\text{Ϣ}}\right)\right)\right)}^{\text{2}}+{\left({\text{𝒷}}^{-\text{1}}\left(\text{ƛ}b\left({\text{ɤ}}_{\text{Ϣ}}\right)\right)\right)}^{\text{2}}=\text{1}-{\left({\text{𝒷}}^{-\text{1}}\left(\text{ƛ}b\left({\text{ɤ}}_{\text{Ϣ}}\right)\right)\right)}^{\text{2}}+{\left({\text{𝒷}}^{-\text{1}}\left(\text{ƛ}b\left({\text{ɤ}}_{\text{Ϣ}}\right)\right)\right)}^{\text{2}}=\text{1}. \end{array}$$

Moreover, since the domain of *ϙ* is the closed unit interval, we conclude that ${\text{ƛ}}_{\text{ϙ}}\text{Ϣ}$ is a Dia‑*PyFS*. It can also be shown that ${\text{Ϣ}}^{{\text{ƛ}}_{\text{ϙ}}}$ is a Dia‑*PyFV*.

**Example 2.** Assume that 𝒷,h,ϙ,σ:[0,1]→[0,∞] characterized by ƛ>0, $\text{𝒷}(\text{𝓉})=-\text{log}{\text{𝓉}}^{\text{2}},h(\text{𝓉})=-\text{log}\left(\text{1}-{\text{𝓉}}^{\text{2}}\right),\text{ϙ}(\text{𝓉})=-\text{log}{\text{𝓉}}^{\text{2}}$ and $\sigma (\text{𝓉})=-\text{log}\left(\text{1}-{\text{𝓉}}^{\text{2}}\right)$. The algebraic operators are then obtained

a) $\text{Ϣ}{⊕}_{\text{ϙ}}L=\left\langle {\left[{\left({\text{ɀ}}_{\text{Ϣ}}\right)}^{\text{2}}+{\left({\text{ɀ}}_{L}\right)}^{\text{2}}-{\left({\text{ɀ}}_{\text{Ϣ}}\right)}^{\text{2}}{\left({\text{ɀ}}_{L}\right)}^{\text{2}}\right]}^{\frac{\text{1}}{\text{2}}},{\text{ɤ}}_{\text{Ϣ}}{\text{ɤ}}_{L};{ℵ}_{\text{Ϣ}}{ℵ}_{L}\right\rangle ,$
b) $\text{Ϣ}{⊕}_{\sigma }L=\left\langle {\left[{\left({\text{ɀ}}_{\text{Ϣ}}\right)}^{\text{2}}+{\left({\text{ɀ}}_{L}\right)}^{\text{2}}-{\left({\text{ɀ}}_{\text{Ϣ}}\right)}^{\text{2}}{\left({\text{ɀ}}_{L}\right)}^{\text{2}}\right]}^{\frac{\text{1}}{\text{2}}},{\text{ɤ}}_{\text{Ϣ}}{\text{ɤ}}_{L};{\left[{\left({ℵ}_{\text{Ϣ}}\right)}^{\text{2}}+{\left({ℵ}_{L}\right)}^{\text{2}}-{\left({ℵ}_{\text{Ϣ}}\right)}^{\text{2}}{\left({ℵ}_{L}\right)}^{\text{2}}\right]}^{\frac{\text{1}}{\text{2}}}\right\rangle$,
c) $\text{Ϣ}{⊗}_{\text{ϙ}}L=\left\langle {\text{ɀ}}_{\text{Ϣ}}{\text{ɀ}}_{L},{\left[{\left({\text{ɤ}}_{\text{Ϣ}}\right)}^{\text{2}}+{\left({\text{ɤ}}_{L}\right)}^{\text{2}}-{\left({\text{ɤ}}_{\text{Ϣ}}\right)}^{\text{2}}{\left({\text{ɤ}}_{L}\right)}^{\text{2}}\right]}^{\frac{\text{1}}{\text{2}}};{ℵ}_{\text{Ϣ}}{ℵ}_{L}\right\rangle ,$
d) $\text{Ϣ}{⊗}_{\sigma }L=\left\langle {\text{ɀ}}_{\text{Ϣ}}{\text{ɀ}}_{L},{\left[{\left({\text{ɤ}}_{\text{Ϣ}}\right)}^{\text{2}}+{\left({\text{ɤ}}_{L}\right)}^{\text{2}}-{\left({\text{ɤ}}_{\text{Ϣ}}\right)}^{\text{2}}{\left({\text{ɤ}}_{L}\right)}^{\text{2}}\right]}^{\frac{\text{1}}{\text{2}}};{\left[{\left({ℵ}_{\text{Ϣ}}\right)}^{\text{2}}+{\left({ℵ}_{L}\right)}^{\text{2}}-{\left({ℵ}_{\text{Ϣ}}\right)}^{\text{2}}{\left({ℵ}_{L}\right)}^{\text{2}}\right]}^{\frac{\text{1}}{\text{2}}}\right\rangle ,$
e) ${\text{ƛ}}_{\text{ϙ}}\text{Ϣ}=\left\langle {\left(\text{1}-{\left(\text{1}-{\left({\text{ɀ}}_{\text{Ϣ}}\right)}^{\text{2}}\right)}^{\text{ƛ}}\right)}^{\frac{\text{1}}{\text{2}}},{\text{ɤ}}_{\text{Ϣ}}^{\text{ƛ}};{ℵ}_{\text{Ϣ}}^{\text{ƛ}}\right\rangle$
f) ${\text{ƛ}}_{\sigma }\text{Ϣ}=\left\langle {\left(\text{1}-{\left(\text{1}-{\left({\text{ɀ}}_{\text{Ϣ}}\right)}^{\text{2}}\right)}^{\text{ƛ}}\right)}^{\frac{\text{1}}{\text{2}}},{\text{ɤ}}_{\text{Ϣ}}^{\text{ƛ}};{\left(\text{1}-{\left(\text{1}-{\left({ℵ}_{\text{Ϣ}}\right)}^{\text{2}}\right)}^{\text{ƛ}}\right)}^{\frac{\text{1}}{\text{2}}}\right\rangle ,$
g) ${\text{Ϣ}}^{{\text{ƛ}}_{\text{ϙ}}}=\left\langle {\text{ɀ}}_{\text{Ϣ}}^{\text{ƛ}},{\left(\text{1}-{\left(\text{1}-{\left({\text{ɤ}}_{\text{Ϣ}}\right)}^{\text{2}}\right)}^{\text{ƛ}}\right)}^{\frac{\text{1}}{\text{2}}};{ℵ}_{\text{Ϣ}}^{\text{ƛ}}\right\rangle ,$
h) ${\text{Ϣ}}^{{\text{ƛ}}_{\sigma }}=\left\langle {\text{ɀ}}_{\text{Ϣ}}^{\text{ƛ}},{\left(\text{1}-{\left(\text{1}-{\left({\text{ɤ}}_{\text{Ϣ}}\right)}^{\text{2}}\right)}^{\text{ƛ}}\right)}^{\frac{\text{1}}{\text{2}}};{\left(\text{1}-{\left(\text{1}-{\left({ℵ}_{\text{Ϣ}}\right)}^{\text{2}}\right)}^{\text{ƛ}}\right)}^{\frac{\text{1}}{\text{2}}}\right\rangle .$

The following theorem presents some fundamental properties of algebraic operations.

**Theorem 2.** Let $\text{Ϣ}=\left\langle {\text{ɀ}}_{\text{Ϣ}},{\text{ɤ}}_{\text{Ϣ}};{ℵ}_{\text{Ϣ}}\right\rangle$, $L=\left\langle {\text{ɀ}}_{L},{\text{ɤ}}_{L};{ℵ}_{L}\right\rangle$ and $T=\left\langle {\text{ɀ}}_{T},{\text{ɤ}}_{T};{ℵ}_{T}\right\rangle$ are three Dia‑*PyFV*s with ƛ,γ>0. Suppose that the additive generator of a continuous Archimedean *𝓉*-norm is 𝒷:[0,1]→[0,∞], and the additive generator of a continuous Archimedean *𝓉*-norm or *𝓉*-conorm is ϙ:[0,1]→[0,∞], with $h(\text{𝓉})=\text{𝒷}\left({\left(\text{1}-{\text{𝓉}}^{\text{2}}\right)}^{\frac{\text{1}}{\text{2}}}\right)$. Then, followings hold such that

1) $\text{Ϣ}{⊕}_{\text{ϙ}}L=L{⊕}_{\text{ϙ}}\text{Ϣ}$,
2) $\text{Ϣ}{⊗}_{\text{ϙ}}L=L{⊗}_{\text{ϙ}}\text{Ϣ}$,
3) $\left(\text{Ϣ}{⊕}_{\text{ϙ}}L\right){⊕}_{\text{ϙ}}T=\text{Ϣ}{⊕}_{\text{ϙ}}\left(L{⊕}_{\text{ϙ}}T\right)$,
4) $\left(\text{Ϣ}{⊗}_{\text{ϙ}}L\right){⊗}_{\text{ϙ}}T=\text{Ϣ}{⊗}_{\text{ϙ}}\left(L{⊗}_{\text{ϙ}}T\right)$,
5) ${\text{ƛ}}_{\text{ϙ}}\left(\text{Ϣ}{⊕}_{\text{ϙ}}L\right)={\text{ƛ}}_{\text{ϙ}}\text{Ϣ}{⊕}_{\text{ϙ}}{\text{ƛ}}_{\text{ϙ}}L$,
6) ${\left(\text{Ϣ}{⊗}_{\text{ϙ}}L\right)}^{{\text{ƛ}}_{\text{ϙ}}}={\text{Ϣ}}^{{\text{ƛ}}_{\text{ϙ}}}{⊗}_{\text{ϙ}}L^{{\text{ƛ}}_{\text{ϙ}}},$,
7) ${\left(\text{Ϣ}{⊗}_{\text{ϙ}}L\right)}^{{\text{ƛ}}_{\text{ϙ}}}={\text{Ϣ}}^{{\text{ƛ}}_{\text{ϙ}}}{⊗}_{\text{ϙ}}L^{{\text{ƛ}}_{\text{ϙ}}},$
8) ${\text{Ϣ}}^{{\text{ƛ}}_{\text{ϙ}}}{⊗}_{\text{ϙ}}{\text{Ϣ}}^{\gamma_{\text{ϙ}}}={\text{Ϣ}}^{{\text{ƛ}}_{\text{ϙ}}+\gamma_{\text{ϙ}}}.$

**Proof.** Obviously, (1), (2), (3) and (4) hold. For (5), we have


$${\text{ƛ}}_{\text{ϙ}}\left(\text{Ϣ}{⊕}_{\text{ϙ}}L\right)$$

$$={\text{ƛ}}_{\text{ϙ}}\left\langle h^{-\text{1}}\left(h\left({\text{ɀ}}_{\text{Ϣ}}\right)+h\left({\text{ɀ}}_{L}\right)\right),{\text{𝒷}}^{-\text{1}}\left(\text{𝒷}\left({\text{ɤ}}_{\text{Ϣ}}\right)+\text{𝒷}\left({\text{ɤ}}_{L}\right)\right);{\text{ϙ}}^{-\text{1}}\left(\text{ϙ}\left({ℵ}_{\text{Ϣ}}\right)+\text{ϙ}\left({ℵ}_{L}\right)\right)\right\rangle$$

$$\begin{array}{cc} & =\left\langle h^{-\text{1}}\left(\text{ƛ}h\left(h^{-\text{1}}\left(h\left({\text{ɀ}}_{\text{Ϣ}}\right)+h\left({\text{ɀ}}_{L}\right)\right)\right)\right),{\text{𝒷}}^{-\text{1}}\left(\text{ƛ}b\left({\text{𝒷}}^{-\text{1}}\left(\text{𝒷}\left({\text{ɤ}}_{\text{Ϣ}}\right)+\text{𝒷}\left({\text{ɤ}}_{L}\right)\right)\right)\right);{\text{ϙ}}^{-\text{1}}\left(\text{ƛ}\text{ϙ}\left({\text{ϙ}}^{-\text{1}}\left(\text{ϙ}\left({ℵ}_{\text{Ϣ}}\right)+\text{ϙ}\left({ℵ}_{L}\right)\right)\right)\right)\right\rangle \\ & =\left\langle h^{-\text{1}}\left(\text{ƛ}h\left({\text{ɀ}}_{\text{Ϣ}}\right)+\text{ƛ}h\left({\text{ɀ}}_{L}\right)\right),{\text{𝒷}}^{-\text{1}}\left(\text{ƛ}b\left({\text{ɤ}}_{\text{Ϣ}}\right)+\text{ƛ}b\left({\text{ɤ}}_{L}\right)\right);{\text{ϙ}}^{-\text{1}}\left(\text{ƛ}\text{ϙ}\left({ℵ}_{\text{Ϣ}}\right)+\text{ƛ}\text{ϙ}\left({ℵ}_{L}\right)\right)\right\rangle \\ & =\left\langle \begin{array}{c} @c@h^{-\text{1}}\left(h\left(h^{-\text{1}}\left(\text{ƛ}h\left({\text{ɀ}}_{\text{Ϣ}}\right)\right)\right)+h\left(h^{-\text{1}}\left(\text{ƛ}h\left({\text{ɀ}}_{L}\right)\right)\right)\right),{\text{𝒷}}^{-\text{1}}(\text{𝒷}({\text{𝒷}}^{-\text{1}}\left(\text{ƛ}b\left({\text{ɤ}}_{\text{Ϣ}}\right)\right)\;\;\\ \;+\text{𝒷}\left({\text{𝒷}}^{-\text{1}}\left(\text{ƛ}b\left({\text{ɤ}}_{L}\right)\right)\right));{\text{ϙ}}^{-\text{1}}\left(\text{ϙ}\left({\text{ϙ}}^{-\text{1}}\left(\text{ƛ}\text{ϙ}\left({ℵ}_{\text{Ϣ}}\right)\right)\right)+\text{ϙ}\left({\text{ϙ}}^{-\text{1}}\left(\text{ƛ}\text{ϙ}\left({ℵ}_{L}\right)\right)\right)\right) \end{array}\;\;\;\;\right\rangle \\ & =\left\langle h^{-\text{1}}\left(h\left({\text{ɀ}}_{\text{ƛ}\text{Ϣ}}\right)+h\left({\text{ɀ}}_{\text{ƛ}L}\right)\right),{\text{𝒷}}^{-\text{1}}\left(\text{𝒷}\left({\text{ɤ}}_{\text{ƛ}\text{Ϣ}}\right)+\text{𝒷}\left({\text{ɤ}}_{\text{ƛ}L}\right)\right);{\text{ϙ}}^{-\text{1}}\left(\text{ϙ}\left({ℵ}_{\text{ƛ}\text{Ϣ}}\right)+\text{ƛ}\text{ϙ}\left({\text{ɤ}}_{\text{ƛ}L}\right)\right)\right\rangle \\ & ={\text{ƛ}}_{\text{ϙ}}\text{Ϣ}{⊕}_{\text{ϙ}}{\text{ƛ}}_{\text{ϙ}}L. \end{array}$$

For (6), we have


$$\begin{array}{cc} \left({\text{ƛ}}_{\text{ϙ}}+\gamma_{\text{ϙ}}\right)\text{Ϣ} & =\left\langle h^{-\text{1}}\left((\text{ƛ}+\gamma )h\left({\text{ɀ}}_{\text{Ϣ}}\right)\right),{\text{𝒷}}^{-\text{1}}\left((\text{ƛ}+\gamma )\text{𝒷}\left({\text{ɤ}}_{\text{Ϣ}}\right)\right);{\text{ϙ}}^{-\text{1}}\left((\text{ƛ}+\gamma )\text{ϙ}\left({ℵ}_{\text{Ϣ}}\right)\right)\right\rangle \\ & =\left\langle h^{-\text{1}}\left(\text{ƛ}h\left({\text{ɀ}}_{\text{Ϣ}}\right)+\gamma h\left({\text{ɀ}}_{\text{Ϣ}}\right)\right),{\text{𝒷}}^{-\text{1}}\left(\text{ƛ}b\left({\text{ɤ}}_{\text{Ϣ}}\right)+\gamma \text{𝒷}\left({\text{ɤ}}_{\text{Ϣ}}\right)\right);{\text{ϙ}}^{-\text{1}}\left(\text{ƛ}\text{ϙ}\left({ℵ}_{\text{Ϣ}}\right)+\gamma \text{ϙ}\left({ℵ}_{\text{Ϣ}}\right)\right)\right\rangle \\ & =\left\langle \begin{array}{c} @c@h^{-\text{1}}\left(h\left(h^{-\text{1}}\left(\text{ƛ}h\left({\text{ɀ}}_{\text{Ϣ}}\right)\right)\right)+h\left(h^{-\text{1}}\left(\gamma h\left({\text{ɀ}}_{\text{Ϣ}}\right)\right)\right)\right),\\ {\text{𝒷}}^{-\text{1}}(\text{𝒷}\left({\text{𝒷}}^{-\text{1}}\left(\text{ƛ}b\left({\text{ɤ}}_{\text{Ϣ}}\right)\right)\right)\;\;+\text{𝒷}\left({\text{𝒷}}^{-\text{1}}\left(\gamma \text{𝒷}\left({\text{ɤ}}_{\text{Ϣ}}\right)\right)\right));{\text{ϙ}}^{-\text{1}}\left(\text{ϙ}\left({\text{ϙ}}^{-\text{1}}\left(\text{ƛ}\text{ϙ}\left({ℵ}_{\text{Ϣ}}\right)\right)\right)+\text{ϙ}\left({\text{ϙ}}^{-\text{1}}\left(\gamma \text{ϙ}\left({ℵ}_{\text{Ϣ}}\right)\right)\right)\right) \end{array}\;\right\rangle \\ & =\left\langle h^{-\text{1}}\left(h\left({\text{ɀ}}_{{\text{ƛ}}_{\text{ϙ}}\text{Ϣ}}\right)+h\left({\text{ɀ}}_{\gamma_{\text{ϙ}}\text{Ϣ}}\right)\right),{\text{𝒷}}^{-\text{1}}\left(\text{𝒷}\left({\text{ɤ}}_{{\text{ƛ}}_{\text{ϙ}}\text{Ϣ}}\right)+\text{𝒷}\left({\text{ɤ}}_{\gamma_{\text{ϙ}}\text{Ϣ}}\right)\right);{\text{ϙ}}^{-\text{1}}\left(\text{ϙ}\left({ℵ}_{{\text{ƛ}}_{\text{ϙ}}\text{Ϣ}}\right)+\text{ϙ}\left({ℵ}_{\gamma_{\text{ϙ}}\text{Ϣ}}\right)\right)\right\rangle \\ & ={\text{ƛ}}_{\text{ϙ}}\text{Ϣ}{⊕}_{\text{ϙ}}\gamma_{\text{ϙ}}\text{Ϣ}. \end{array}$$

For (7), we have


$${\left(\text{Ϣ}{⊗}_{\text{ϙ}}L\right)}^{{\text{ƛ}}_{\text{ϙ}}}=\left\langle {\text{𝒷}}^{-\text{1}}\left(\text{ƛ}b\left({\text{ɀ}}_{\text{Ϣ}{⊗}_{\text{ϙ}}L}\right)\right),h^{-\text{1}}\left(\text{ƛ}h\left({\text{ɤ}}_{\text{Ϣ}{⊗}_{\text{ϙ}}L}\right)\right);{\text{ϙ}}^{-\text{1}}\left(\text{ƛ}\text{ϙ}\left({ℵ}_{\text{Ϣ}{⊗}_{\text{ϙ}}L}\right)\right)\right\rangle$$

$$=\langle {\text{𝒷}}^{-\text{1}}\left(\text{ƛ}b\left({\text{𝒷}}^{-\text{1}}\left(\text{𝒷}\left({\text{ɀ}}_{\text{Ϣ}}\right)+\text{𝒷}\left({\text{ɀ}}_{L}\right)\right)\right)\right),h^{-\text{1}}\left(\text{ƛ}h\left(h^{-\text{1}}\left(h\left({\text{ɤ}}_{\text{Ϣ}}\right)+h\left({\text{ɤ}}_{L}\right)\right)\right)\right)\;;$$

$$\;{\text{ϙ}}^{-\text{1}}\left(\text{ƛ}\text{ϙ}\left({\text{ϙ}}^{-\text{1}}\left(\text{ϙ}\left({ℵ}_{\text{Ϣ}}\right)+\text{ϙ}\left({ℵ}_{L}\right)\right)\right)\right)\rangle$$

$$=\langle {\text{𝒷}}^{-\text{1}}\left(\text{ƛ}b\left({\text{ɀ}}_{\text{Ϣ}}\right)+\text{ƛ}b\left({\text{ɀ}}_{L}\right)\right),h^{-\text{1}}\left(\text{ƛ}h\left({\text{ɤ}}_{\text{Ϣ}}\right)+\text{ƛ}h\left({\text{ɤ}}_{L}\right)\right)\;;\;{\text{ϙ}}^{-\text{1}}\left(\text{ƛ}\text{ϙ}\left({ℵ}_{\text{Ϣ}}\right)+\text{ƛ}\text{ϙ}\left({ℵ}_{L}\right)\right)\rangle$$

$$=\langle {\text{𝒷}}^{-\text{1}}\left(\text{𝒷}\left({\text{𝒷}}^{-\text{1}}\left(\text{ƛ}b\left({\text{ɀ}}_{\text{Ϣ}}\right)\right)\right)+\text{𝒷}\left({\text{𝒷}}^{-\text{1}}\left(\text{ƛ}b\left({\text{ɀ}}_{L}\right)\right)\right)\right),h^{-\text{1}}(h\left(h^{-\text{1}}\left(\text{ƛ}h\left({\text{ɤ}}_{\text{Ϣ}}\right)\right)\right)\;\;$$

$$+\;h\left(h^{-\text{1}}\left(\text{ƛ}h\left({\text{ɤ}}_{L}\right)\right)\right);{\text{ϙ}}^{-\text{1}}\left(\text{ϙ}\left({\text{ϙ}}^{-\text{1}}\left(\text{ƛ}\text{ϙ}\left({ℵ}_{\text{Ϣ}}\right)\right)\right)+\text{ϙ}\left({\text{ϙ}}^{-\text{1}}\left(\text{ƛ}\text{ϙ}\left({ℵ}_{L}\right)\right)\right)\right\rangle$$

$$=\langle {\text{𝒷}}^{-\text{1}}\left(\text{𝒷}\left({\text{ɀ}}_{{\text{Ϣ}}^{\text{ƛ}}\text{ϙ}}\right)+\text{𝒷}\left({\text{ɀ}}_{L^{\text{ƛ}\text{ϙ}}}\right)\right),h^{-\text{1}}\left(h\left({\text{ɤ}}_{{\text{Ϣ}}^{\text{ƛ}}\text{ϙ}}\right)+h\left({\text{ɤ}}_{L^{\text{ƛ}}\text{ϙ}}\right)\right)\;;\;{\text{ϙ}}^{-\text{1}}\left(\text{ϙ}\left({ℵ}_{{\text{Ϣ}}^{\text{ƛ}}\text{ϙ}}\right)+\text{ϙ}\left({ℵ}_{L^{\text{ƛ}\text{ϙ}}}\right)\right)\rangle$$

$$={\text{Ϣ}}^{{\text{ƛ}}_{\text{ϙ}}}{⊗}_{\text{ϙ}}L^{{\text{ƛ}}_{\text{ϙ}}}.$$

For (8), we have


$${\text{Ϣ}}^{{\text{ƛ}}_{\text{ϙ}}+\gamma_{\text{ϙ}}}$$

$$=\left\langle {\text{𝒷}}^{-\text{1}}\left((\text{ƛ}+\gamma )\text{𝒷}\left({\text{ɀ}}_{\text{Ϣ}}\right)\right),h^{-\text{1}}\left((\text{ƛ}+\gamma )h\left({\text{ɤ}}_{\text{Ϣ}}\right)\right)\;;{\text{ϙ}}^{-\text{1}}\left((\text{ƛ}+\gamma )\text{ϙ}\left({ℵ}_{\text{Ϣ}}\right)\right)\right\rangle$$

$$=\langle {\text{𝒷}}^{-\text{1}}\left(\text{ƛ}b\left({\text{ɀ}}_{\text{Ϣ}}\right)+\gamma \text{𝒷}\left({\text{ɀ}}_{\text{Ϣ}}\right)\right),h^{-\text{1}}\left(\text{ƛ}h\left({\text{ɤ}}_{\text{Ϣ}}\right)+\gamma h\left({\text{ɤ}}_{\text{Ϣ}}\right)\right)\;;\;{\text{ϙ}}^{-\text{1}}\left(\text{ƛ}\text{ϙ}\left({ℵ}_{\text{Ϣ}}\right)+\gamma \text{ϙ}\left({ℵ}_{\text{Ϣ}}\right)\right)\rangle$$

$$=\langle {\text{𝒷}}^{-\text{1}}\left(\text{𝒷}\left({\text{𝒷}}^{-\text{1}}\left(\text{ƛ}b\left({\text{ɀ}}_{\text{Ϣ}}\right)\right)\right)+\text{𝒷}\left({\text{𝒷}}^{-\text{1}}\left(\gamma \text{𝒷}\left({\text{ɀ}}_{\text{Ϣ}}\right)\right)\right)\right),h^{-\text{1}}(h\left(h^{-\text{1}}\left(\text{ƛ}h\left({\text{ɤ}}_{\text{Ϣ}}\right)\right)\right)\;\;$$

$$\;\;+\;h\left(h^{-\text{1}}\left(\gamma h\left({\text{ɤ}}_{\text{Ϣ}}\right)\right)\right));{\text{ϙ}}^{-\text{1}}\left(\text{ϙ}\left({\text{ϙ}}^{-\text{1}}\left(\text{ƛ}\text{ϙ}\left({ℵ}_{\text{Ϣ}}\right)\right)\right)+\text{ϙ}\left({\text{ϙ}}^{-\text{1}}\left(\gamma \text{ϙ}\left({ℵ}_{\text{Ϣ}}\right)\right)\right)\right)\rangle$$

$$=\left\langle {\text{𝒷}}^{-\text{1}}\left(\text{𝒷}\left({\text{ɀ}}_{{\text{Ϣ}}^{\text{ƛ}}}\right)+\text{𝒷}\left({\text{ɀ}}_{{\text{Ϣ}}^{\gamma }}\right)\right),h^{-\text{1}}\left(h\left({\text{ɤ}}_{{\text{Ϣ}}^{\text{ƛ}}}\right)+h\left({\text{ɤ}}_{\text{Ϣ}\gamma }\right)\right);{\text{ϙ}}^{-\text{1}}\left(\text{ϙ}\left({ℵ}_{{\text{Ϣ}}^{\text{ƛ}}}\right)+\text{ϙ}\left({ℵ}_{{\text{Ϣ}}^{\gamma }}\right)\right)\right\rangle$$

$$={\text{Ϣ}}^{{\text{ƛ}}_{\text{ϙ}}}{⊗}_{\text{ϙ}}{\text{Ϣ}}^{\gamma_{\text{ϙ}}}.$$

## 6. Aggregation operators via Dia‑*PyN*s

In data consolidation, especially in decision-making scenarios, the goal of aggregation is to generate a data summary before arriving at a final decision. This section discusses the Dia‑*PyF*-weighted averaging and geometric aggregation operators. It is important to note that Dia‑*PyFV*s on *E* are denoted by Dia‑*PyFV(E)*. Additionally, we calculate the Euclidean distance measure between Dia‑*PyFV*s.

### 6.1. Diamond Pythagorean fuzzy weighted averaging aggregation operators

**Definition 17.** Let $\left\{{\text{Ϣ}}_{i}=\left\langle {\text{ɀ}}_{{\text{Ϣ}}_{i}},{\text{ɤ}}_{{\text{Ϣ}}_{i}},;{ℵ}_{{\text{Ϣ}}_{i}}\right\rangle :i=\text{1},\ldots ,n\right\}$ be the set of Dia‑*PyFV*s. Suppose that the additive generator of a continuous Archimedean *𝓉*-norm is 𝒷:[0,1]→[0,∞], and the additive generator of a continuous Archimedean *𝓉*-norm or *𝓉*-conorm is ϙ:[0,1]→[0,∞], with $h(\text{𝓉})=\text{𝒷}\left({\left(\text{1}-{\text{𝓉}}^{\text{2}}\right)}^{\frac{\text{1}}{\text{2}}}\right)$. Then, Dia‑*PyFWAA* operator with mapping Dia-PyFWAA:Dia-PyFV(E)→Dia-PyFV(E) is computed as follows


$$\text{Dia-}PyFWA{A_{\text{ϙ}}}_{\;}\left({\text{Ϣ}}_{\text{1}},{\text{Ϣ}}_{\text{2}},{\text{Ϣ}}_{\text{3}},\ldots \ldots \ldots ,\;{\text{Ϣ}}_{n}\right)=(\text{ϙ}){⨁}_{i=\text{1}}^{n}\;\varpi_{i\text{ϙ}}{\text{Ϣ}}_{i},$$

with weight vector $\varpi {=\left(\varpi_{\text{1}},\;\varpi_{\text{2}},\;\varpi_{\text{3}},\ldots ,\varpi_{n}\right)}^{T}$ with $\text{0}\leq \varpi_{j}\leq \text{1}$ and $\sum_{j=\text{1}}^{n}\varpi_{j}=\text{1}$.

**Theorem 3.** Let $\left\{{\text{Ϣ}}_{i}=\left\langle {\text{ɀ}}_{{\text{Ϣ}}_{i}},{\text{ɤ}}_{{\text{Ϣ}}_{i}},;{ℵ}_{{\text{Ϣ}}_{i}}\right\rangle :i=\text{1},\ldots ,n\right\}$ be the set of Dia‑*PyFV*s. Suppose that the additive generator of a continuous Archimedean *𝓉*-norm is 𝒷:[0,1]→[0,∞], and the additive generator of a continuous Archimedean *𝓉*-norm or *𝓉*-conorm is ϙ:[0,1]→[0,∞], with $h(\text{𝓉})=\text{𝒷}\left({\left(\text{1}-{\text{𝓉}}^{\text{2}}\right)}^{\frac{\text{1}}{\text{2}}}\right)$. If Dia‑*PyFWAA* operator is defined with the help of this transformation Dia-PyFWAA:Dia-PyFV(E)→Dia-PyFV(E), then $\text{Dia}-PyFWA{A_{\text{ϙ}}}_{\;}\left({\text{Ϣ}}_{\text{1}},{\text{Ϣ}}_{\text{2}},{\text{Ϣ}}_{\text{3}},\ldots \ldots \ldots ,\;{\text{Ϣ}}_{i}\right)$ is Dia‑*PyFV* and we have


$$\text{Dia-}PyFWA{A_{\text{ϙ}}}_{\;}\left({\text{Ϣ}}_{\text{1}},{\text{Ϣ}}_{\text{2}},{\text{Ϣ}}_{\text{3}},\ldots \ldots \ldots ,\;{\text{Ϣ}}_{n}\right)=\left\langle h^{-\text{1}}\left(\sum_{i=\text{1}}^{n}\;\;\varpi_{i}h\left({\text{ɀ}}_{{\text{Ϣ}}_{i}}\right)\right),{\text{𝒷}}^{-\text{1}}\left(\sum_{i=\text{1}}^{n}\;\;\varpi_{i}\text{𝒷}\left({\text{ɤ}}_{{\text{Ϣ}}_{i}}\right)\right);{\text{ϙ}}^{-\text{1}}\left(\sum_{i=\text{1}}^{n}\;\;\varpi_{i}\text{ϙ}\left({ℵ}_{{\text{Ϣ}}_{i}}\right)\right)\right\rangle ,$$

with weight vector $\varpi {=\left(\varpi_{\text{1}},\;\varpi_{\text{2}},\;\varpi_{\text{3}},\ldots ,\varpi_{n}\right)}^{T}$ with $\text{0}\leq \varpi_{j}\leq \text{1}$ and $\sum_{j=\text{1}}^{n}\varpi_{j}=\text{1}$.

**Proof.** By hypothesis, $\text{Dia}-PyFWA{A_{\text{ϙ}}}_{\;}\left({\text{Ϣ}}_{\text{1}},{\text{Ϣ}}_{\text{2}},{\text{Ϣ}}_{\text{3}},\ldots \ldots \ldots ,\;{\text{Ϣ}}_{n}\right)$ is a Dia‑*PyFV*. By utilizing mathematical induction, it can be seen that the second part is also true. If *n = 2*, then we have


$$\text{Dia-}PyFWA{A_{\text{ϙ}}}_{\;}\left({\text{Ϣ}}_{\text{1}},{\text{Ϣ}}_{\text{2}}\right)=\varpi_{\text{1}\text{ϙ}}{\text{Ϣ}}_{\text{1}}{⊕}_{\text{ϙ}}\varpi_{\text{2}\text{ϙ}}{\text{Ϣ}}_{\text{2}}$$

$$=\langle h^{-\text{1}}\left(h\left({\text{ɀ}}_{\varpi_{\text{1}\text{ϙ}}{\text{Ϣ}}_{\text{1}}}\right)+h\left({\text{ɀ}}_{\varpi_{\text{2}\text{ϙ}}{\text{Ϣ}}_{\text{2}}}\right)\right),{\text{𝒷}}^{-\text{1}}\left(\text{𝒷}\left({\text{ɤ}}_{\varpi_{\text{1}\text{ϙ}}{\text{Ϣ}}_{\text{1}}}\right)+\text{𝒷}\left({\text{ɤ}}_{\varpi_{\text{2}\text{ϙ}}{\text{Ϣ}}_{\text{2}}}\right)\right)\;$$

$$\;{\text{ϙ}}^{-\text{1}}\left(\text{ϙ}\left({ℵ}_{\varpi_{\text{1}\text{ϙ}}{\text{Ϣ}}_{\text{1}}}\right)+\text{ϙ}\left({ℵ}_{\varpi_{\text{2}\text{ϙ}}{\text{Ϣ}}_{\text{2}}}\right)\right)\rangle$$

$$=\langle h^{-\text{1}}\left(h\left(h^{-\text{1}}\left(\varpi_{\text{1}}h\left({\text{ɀ}}_{{\text{Ϣ}}_{\text{1}}}\right)\right)\right)+h\left(h^{-\text{1}}\left(\varpi_{\text{1}}h\left({\text{ɀ}}_{{\text{Ϣ}}_{\text{1}}}\right)\right)\right)\right)\;,$$

$${\text{𝒷}}^{-\text{1}}\left(\text{𝒷}\left({\text{𝒷}}^{-\text{1}}\left(\varpi_{\text{1}}\text{𝒷}\left({\text{ɤ}}_{{\text{Ϣ}}_{\text{1}}}\right)\right)\right)+\text{𝒷}\left({\text{𝒷}}^{-\text{1}}\left(\varpi_{\text{2}}\text{𝒷}\left({\text{ɤ}}_{{\text{Ϣ}}_{\text{2}}}\right)\right)\right)\right);$$

$${\text{ϙ}}^{-\text{1}}\left(\text{ϙ}\left({\text{ϙ}}^{-\text{1}}\left(\varpi_{\text{1}}\text{ϙ}\left({ℵ}_{{\text{Ϣ}}_{\text{1}}}\right)\right)\right)+\text{ϙ}\left({\text{ϙ}}^{-\text{1}}\left(\varpi_{\text{2}}\text{ϙ}\left({ℵ}_{{\text{Ϣ}}_{\text{2}}}\right)\right)\right)\right\rangle$$

$$=\langle h^{-\text{1}}\left(\varpi_{\text{1}}h\left({\text{ɀ}}_{{\text{Ϣ}}_{\text{1}}}\right)+\varpi_{\text{2}}h\left({\text{ɀ}}_{{\text{Ϣ}}_{\text{2}}}\right)\right),{\text{𝒷}}^{-\text{1}}\left(\varpi_{\text{1}}\text{𝒷}\left({\text{ɤ}}_{{\text{Ϣ}}_{\text{1}}}\right)+\varpi_{\text{2}}\text{𝒷}\left({\text{ɤ}}_{{\text{Ϣ}}_{\text{2}}}\right)\right)\;;$$

$$\;{\text{ϙ}}^{-\text{1}}\left(\varpi_{\text{1}}\text{ϙ}\left({ℵ}_{{\text{Ϣ}}_{\text{1}}}\right)+\varpi_{\text{2}}\text{𝒷}\left({ℵ}_{{\text{Ϣ}}_{\text{2}}}\right)\right)\rangle$$

$$=\langle h^{-\text{1}}\left(\sum_{j=\text{1}}^{\text{2}}\;\;\varpi_{j}h\left({\text{ɀ}}_{{\text{Ϣ}}_{j}}\right)\right),{\text{𝒷}}^{-\text{1}}\left(\sum_{j=\text{1}}^{\text{2}}\;\;\varpi_{j}\text{𝒷}\left({\text{ɤ}}_{{\text{Ϣ}}_{j}}\right)\right);\;{\text{ϙ}}^{-\text{1}}\left(\sum_{j=\text{1}}^{\text{2}}\;\;\varpi_{j}\text{ϙ}\left({ℵ}_{{\text{Ϣ}}_{j}}\right)\right)\rangle \;$$

Let us temporarily assume that the following expression hold such that


$$A_{n-\text{1}}=\text{Dia-}PyFWA{A_{\text{ϙ}}}_{\;}\left({\text{Ϣ}}_{\text{1}},\ldots ,{\text{Ϣ}}_{n-\text{1}}\right)=\left\langle h^{-\text{1}}\left(\sum_{j=\text{1}}^{n-\text{1}}\;\;\varpi_{j}h\left({\text{ɀ}}_{{\text{Ϣ}}_{j}}\right)\right),{\text{𝒷}}^{-\text{1}}\left(\sum_{j=\text{1}}^{n-\text{1}}\;\;\varpi_{j}\text{𝒷}\left({\text{ɤ}}_{{\text{Ϣ}}_{j}}\right)\right);{\text{ϙ}}^{-\text{1}}\left(\sum_{j=\text{1}}^{n-\text{1}}\;\;\varpi_{j}\text{ϙ}\left({ℵ}_{{\text{Ϣ}}_{j}}\right)\right)\right\rangle .$$

We now have


$$\text{Dia-}PyFWA{A_{\text{ϙ}}}_{\;}\left({\text{Ϣ}}_{\text{1}},\ldots ,{\text{Ϣ}}_{n}\right)=A_{n-\text{1}}{⊕}_{\text{ϙ}}\varpi_{n_{\text{ϙ}}}{\text{Ϣ}}_{n}=\left\langle h^{-\text{1}}\left(\sum_{j=\text{1}}^{n-\text{1}}\;\;\varpi_{j}h\left({\text{ɀ}}_{{\text{Ϣ}}_{j}}\right)\right),{\text{𝒷}}^{-\text{1}}\left(\sum_{j=\text{1}}^{n-\text{1}}\;\;\varpi_{j}\text{𝒷}\left({\text{ɤ}}_{{\text{Ϣ}}_{j}}\right)\right);{\text{ϙ}}^{-\text{1}}\left(\sum_{j=\text{1}}^{n-\text{1}}\;\;\varpi_{j}\text{ϙ}\left({ℵ}_{{\text{Ϣ}}_{j}}\right)\right)\right\rangle$$

$${⊕}_{\text{ϙ}}\left\langle h^{-\text{1}}\left(\varpi_{n}\left({\text{ɀ}}_{{\text{Ϣ}}_{n}}\right)\right),{\text{𝒷}}^{-\text{1}}\left(\varpi_{n}\text{𝒷}\left({\text{ɤ}}_{{\text{Ϣ}}_{n}}\right)\right);{\text{ϙ}}^{-\text{1}}\left(\varpi_{n}\text{ϙ}\left({ℵ}_{{\text{Ϣ}}_{n}}\right)\right)\right\rangle =\langle h^{-\text{1}}\left(h\left(h^{-\text{1}}\left(\sum_{j=\text{1}}^{n-\text{1}}\;\;\varpi_{j}h\left({\text{ɀ}}_{{\text{Ϣ}}_{j}}\right)\right)\right)+h\left(h^{-\text{1}}\left(\varpi_{n}h\left({\text{ɀ}}_{{\text{Ϣ}}_{n}}\right)\right)\right)\right),\;$$

$$\;{\text{𝒷}}^{-\text{1}}\left(\text{𝒷}\left({\text{𝒷}}^{-\text{1}}\left(\sum_{j=\text{1}}^{n-\text{1}}\;\;\varpi_{j}\text{𝒷}\left({\text{ɤ}}_{{\text{Ϣ}}_{j}}\right)\right)\right)+{\text{𝒷}}^{-\text{1}}\left(\varpi_{n}\text{𝒷}\left({\text{ɤ}}_{{\text{Ϣ}}_{n}}\right)\right)\right));$$

$$\begin{array}{cc} & \;{\text{ϙ}}^{-\text{1}}\left(\text{ϙ}\left({\text{ϙ}}^{-\text{1}}\left(\sum_{j=\text{1}}^{n-\text{1}}\;\;\varpi_{j}\text{ϙ}\left({\text{ɤ}}_{{\text{Ϣ}}_{j}}\right)\right)\right)+\text{ϙ}\left({\text{ϙ}}^{-\text{1}}\left(\varpi_{n}\text{ϙ}\left({\text{ɤ}}_{{\text{Ϣ}}_{n}}\right)\right)\right)\right)\rangle \\ & \;=\langle h^{-\text{1}}\left(\sum_{j=\text{1}}^{n-\text{1}}\;\;\varpi_{j}h\left({\text{ɀ}}_{{\text{Ϣ}}_{j}}\right)+\varpi_{n}h\left({\text{ɀ}}_{{\text{Ϣ}}_{n}}\right)\right),{\text{𝒷}}^{-\text{1}}\left(\sum_{j=\text{1}}^{n-\text{1}}\;\;\varpi_{j}\text{𝒷}\left({\text{ɤ}}_{{\text{Ϣ}}_{j}}\right)+\varpi_{n}\text{𝒷}\left({\text{ɤ}}_{{\text{Ϣ}}_{n}}\right)\right)\;;\; \end{array}$$

$$\;{\text{ϙ}}^{-\text{1}}\left(\sum_{j=\text{1}}^{n-\text{1}}\;\;\varpi_{j}\text{ϙ}\left({ℵ}_{{\text{Ϣ}}_{j}}\right)+\varpi_{n}\text{ϙ}\left({ℵ}_{{\text{Ϣ}}_{n}}\right)\right)\rangle =\left\langle h^{-\text{1}}\left(\sum_{i=\text{1}}^{n}\;\;\varpi_{i}h\left({\text{ɀ}}_{{\text{Ϣ}}_{i}}\right)\right),{\text{𝒷}}^{-\text{1}}\left(\sum_{i=\text{1}}^{n}\;\;\varpi_{i}\text{𝒷}\left({\text{ɤ}}_{{\text{Ϣ}}_{i}}\right)\right);{\text{ϙ}}^{-\text{1}}\left(\sum_{i=\text{1}}^{n}\;\;\varpi_{i}\text{ϙ}\left({ℵ}_{{\text{Ϣ}}_{i}}\right)\right)\right\rangle .$$

That concludes the proof.

**Corollary 1.** Assume that 𝒷,h,ϙ,σ:[0,1]→[0,∞] characterized by, $\text{𝒷}\left(\text{𝓉}\right)=-\text{log}{\text{𝓉}}^{\text{2}},h\left(\text{𝓉}\right)=\;-\text{log}\left(\text{1}-{\text{𝓉}}^{\text{2}}\right),\text{ϙ}(\text{𝓉})=-\text{log}{\text{𝓉}}^{\text{2}}$ and $\sigma (\text{𝓉})=-\text{log}\left(\text{1}-{\text{𝓉}}^{\text{2}}\right)$. The algebraic Dia‑*PyFWAA* operators are then obtained such that


$$\text{Dia-}PyFWA{A_{\text{ϙ}}}_{\;}\left({\text{Ϣ}}_{\text{1}},\ldots ,{\text{Ϣ}}_{n}\right)=\left\langle {\left(\text{1}-\prod_{i=\text{1}}^{n}\;\;{\left(\text{1}-{\left({\text{ɀ}}_{{\text{Ϣ}}_{i}}\right)}^{\text{2}}\right)}^{\varpi_{i}}\right)}^{\frac{\text{1}}{\text{2}}},\prod_{i=\text{1}}^{n}\;{\text{ɤ}}_{{\text{Ϣ}}_{i}}^{\varpi_{i}};\prod_{i=\text{1}}^{n}\;\;{ℵ}_{{\text{Ϣ}}_{i}}^{\varpi_{i}}\right\rangle ,$$

and


$$\text{Dia-}PyFWA{A_{\sigma }}_{\;}\left({\text{Ϣ}}_{\text{1}},\ldots ,{\text{Ϣ}}_{n}\right)=\left\langle {\left(\text{1}-\prod_{i=\text{1}}^{n}\;\;{\left(\text{1}-{\left({\text{ɀ}}_{{\text{Ϣ}}_{i}}\right)}^{\text{2}}\right)}^{\varpi_{i}}\right)}^{\frac{\text{1}}{\text{2}}},\prod_{i=\text{1}}^{n}\;\;{\text{ɤ}}_{{\text{Ϣ}}_{i}}^{\varpi_{i}};{\left(\text{1}-\prod_{i=\text{1}}^{n}\;\;{\left(\text{1}-{\left({ℵ}_{{\text{Ϣ}}_{i}}\right)}^{\text{2}}\right)}^{\varpi_{i}}\right)}^{\frac{\text{1}}{\text{2}}}\right\rangle .$$

### 6.2. Diamond Pythagorean fuzzy weighted geometric aggregation operators

**Definition 18.** Let $\left\{{\text{Ϣ}}_{i}=\left\langle {\text{ɀ}}_{{\text{Ϣ}}_{i}},{\text{ɤ}}_{{\text{Ϣ}}_{i}},;{ℵ}_{{\text{Ϣ}}_{i}}\right\rangle :i=\text{1},\ldots ,n\right\}$ be the set of Dia‑*PyFV*s. Suppose that the additive generator of a continuous Archimedean *𝓉*-norm is 𝒷:[0,1]→[0,∞], and the additive generator of a continuous Archimedean *𝓉*-norm or *𝓉*-conorm is ϙ:[0,1]→[0,∞], with h(𝓉)=𝒷(1−𝓉). Then, diamond Pythagorean fuzzy weighted geometric aggregation (Dia‑*PyFWGA*) operator with mapping Dia-PyFWGA:Dia-PyFV(E)→Dia-PyFV(E) is computed as follows


$$\text{Dia-}PyFWG{A_{\text{ϙ}}}_{\;}\left({\text{Ϣ}}_{\text{1}},{\text{Ϣ}}_{\text{2}},{\text{Ϣ}}_{\text{3}},\ldots ,\;{\text{Ϣ}}_{n}\right)=(\text{ϙ}){⨁}_{i=\text{1}}^{n}\;{\text{Ϣ}}_{i}^{\varpi_{i\text{ϙ}}},$$

with weight vector $\varpi {=\left(\varpi_{\text{1}},\;\varpi_{\text{2}},\;\varpi_{\text{3}},\ldots ,\varpi_{n}\right)}^{T}$ with $\text{0}\leq \varpi_{j}\leq \text{1}$ and $\sum_{j=\text{1}}^{n}\varpi_{j}=\text{1}$.

**Theorem 4.** Let $\left\{{\text{Ϣ}}_{i}=\left\langle {\text{ɀ}}_{{\text{Ϣ}}_{i}},{\text{ɤ}}_{{\text{Ϣ}}_{i}},;{ℵ}_{{\text{Ϣ}}_{i}}\right\rangle :i=\text{1},\ldots ,n\right\}$ be the set of Dia‑*PyFV*s. Suppose that the additive generator of a continuous Archimedean *𝓉*-norm is 𝒷:[0,1]→[0,∞], and the additive generator of a continuous Archimedean *𝓉*-norm or *𝓉*-conorm is ϙ:[0,1]→[0,∞], with $h(\text{𝓉})=\text{𝒷}\left({\left(\text{1}-{\text{𝓉}}^{\text{2}}\right)}^{\frac{\text{1}}{\text{2}}}\right)$. If Dia‑*PyFWGA* operator is defined with the help of this transformation Dia-PyFWGA:Dia-PyFV(E)→Dia-PyFV(E), then $\text{Dia-}PyFWG{A_{\text{ϙ}}}_{\;}\left({\text{Ϣ}}_{\text{1}},{\text{Ϣ}}_{\text{2}},{\text{Ϣ}}_{\text{3}},\ldots ,\;{\text{Ϣ}}_{n}\right)$ is Dia‑*PyFV* and we have


$$\text{Dia-}PyFWG{A_{\text{ϙ}}}_{\;}\left({\text{Ϣ}}_{\text{1}},{\text{Ϣ}}_{\text{2}},{\text{Ϣ}}_{\text{3}},\ldots \ldots \ldots ,\;{\text{Ϣ}}_{n}\right)=\left\langle {\text{𝒷}}^{-\text{1}}\left(\sum_{i=\text{1}}^{n}\;\;\varpi_{i}\text{𝒷}\left({\text{ɀ}}_{{\text{Ϣ}}_{i}}\right)\right),h^{-\text{1}}\left(\sum_{i=\text{1}}^{n}\;\;\varpi_{i}h\left({\text{ɤ}}_{{\text{Ϣ}}_{i}}\right)\right);{\text{ϙ}}^{-\text{1}}\left(\sum_{i=\text{1}}^{n}\;\;\varpi_{i}\text{ϙ}\left({ℵ}_{{\text{Ϣ}}_{i}}\right)\right)\right\rangle ,$$

with weight vector $\varpi {=\left(\varpi_{\text{1}},\;\varpi_{\text{2}},\;\varpi_{\text{3}},\ldots ,\varpi_{n}\right)}^{T}$ with $\text{0}\leq \varpi_{j}\leq \text{1}$ and $\sum_{j=\text{1}}^{n}\varpi_{j}=\text{1}$.

**Proof.** By using same arguments like Theorem 4, it can be proven.

**Corollary 2.** Assume that 𝒷,h,ϙ,σ:[0,1]→[0,∞] characterized by, $\text{𝒷}(\text{𝓉})=-\text{log}{\text{𝓉}}^{\text{2}},h(\text{𝓉})=$ $-\text{log}\left(\text{1}-{\text{𝓉}}^{\text{2}}\right),\text{ϙ}(\text{𝓉})=-\text{log}{\text{𝓉}}^{\text{2}}$ and *σ(*𝓉*) = -log (1–*𝓉**^*2*^). The algebraic Dia‑*PyFWGA* operators are then obtained such that


$$\text{Dia-}PyFWG{A_{\text{ϙ}}}_{\;}\left({\text{Ϣ}}_{\text{1}},\ldots ,{\text{Ϣ}}_{n}\right)=\left\langle \prod_{i=\text{1}}^{n}\;{\text{ɀ}}_{{\text{Ϣ}}_{i}}^{\varpi_{i}},{\left(\text{1}-\prod_{i=\text{1}}^{n}\;\;{\left(\text{1}-{\left({\text{ɤ}}_{{\text{Ϣ}}_{i}}\right)}^{\text{2}}\right)}^{\varpi_{i}}\right)}^{\frac{\text{1}}{\text{2}}};\prod_{i=\text{1}}^{n}\;\;{ℵ}_{{\text{Ϣ}}_{i}}^{\varpi_{i}}\right\rangle ,$$

and


$$\text{Dia-}PyFWG{A_{\sigma }}_{\;}\left({\text{Ϣ}}_{\text{1}},\ldots ,{\text{Ϣ}}_{n}\right)=\left\langle \prod_{i=\text{1}}^{n}\;{\text{ɀ}}_{{\text{Ϣ}}_{i}}^{\varpi_{i}},{\left(\text{1}-\prod_{i=\text{1}}^{n}\;\;{\left(\text{1}-{\left({\text{ɤ}}_{{\text{Ϣ}}_{i}}\right)}^{\text{2}}\right)}^{\varpi_{i}}\right)}^{\frac{\text{1}}{\text{2}}};{\left(\text{1}-\prod_{i=\text{1}}^{n}\;\;{\left(\text{1}-{\left({ℵ}_{{\text{Ϣ}}_{i}}\right)}^{\text{2}}\right)}^{\varpi_{i}}\right)}^{\frac{\text{1}}{\text{2}}}\right\rangle .$$

### 6.3. Distance measures via Dia‑*PyFSs*

The next outcomes are introduced for representing different distances over Dia‑*PyFSs* via Subsection 3 approach.

**Definition 19.** Let *d* be a cardinality of *E*. Then normalized Euclidean distance for two Dia‑*PyFSs* ${\text{Ϣ}}_{{ℵ}_{\text{1}}}^{py}$ and ${\text{Ϣ}}_{{ℵ}_{\text{2}}}^{py}$ is defined as


$$H_{\text{2}}^{q}\left({\text{Ϣ}}_{{ℵ}_{\text{1}}}^{py},\;{\text{Ϣ}}_{{ℵ}_{\text{2}}}^{py}\right)=\frac{\text{1}}{\text{2}}\times \left(\frac{\text{1}}{d}\sum_{\rho \in E}\frac{\left|{ℵ}_{\text{1}}-{ℵ}_{\text{2}}\right|}{\text{2}}+\sqrt[{q}]{\frac{\text{1}}{\text{2}d}\sum_{\rho \in E}\left({\left|{\left({\text{ɀ}}_{{\text{Ϣ}}_{\text{1}}}(\rho )\right)}^{\text{2}}-{\left({\text{ɀ}}_{{\text{Ϣ}}_{\text{2}}}(\rho )\right)}^{\text{2}}\right|}^{q}+{\left|{\left({\text{ɤ}}_{{\text{Ϣ}}_{\text{1}}}(\rho )\right)}^{\text{2}}-{\left({\text{ɤ}}_{{\text{Ϣ}}_{\text{2}}}(\rho )\right)}^{\text{2}}\right|}^{q}\right)}\;\right),$$

where *q = 1, 2*. If *q = 1* and *q = 2*, then distance $H_{\text{2}}^{\text{1}}\left({\text{Ϣ}}_{{ℵ}_{\text{1}}}^{py},\;{\text{Ϣ}}_{{ℵ}_{\text{2}}}^{py}\right)$ and $H_{\text{2}}^{\text{2}}\left({\text{Ϣ}}_{{ℵ}_{\text{1}}}^{py},\;{\text{Ϣ}}_{{ℵ}_{\text{2}}}^{py}\right)$ are known as Hamming distance and Euclidean distance for Dia‑*PyFSs*, respectively.

**Definition 20.** Let *d* be a cardinality of *E*. Then normalized Euclidean distance for two Dia‑*PyFSs* ${\text{Ϣ}}_{{ℵ}_{\text{1}}}^{py}$ and ${\text{Ϣ}}_{{ℵ}_{\text{2}}}^{py}$ is defined as


$$H_{\text{3}}^{q}\left({\text{Ϣ}}_{{ℵ}_{\text{1}}}^{py},\;{\text{Ϣ}}_{{ℵ}_{\text{2}}}^{py}\right)=\frac{\text{1}}{\text{2}}\times \left(\frac{\text{1}}{d}\sum_{\rho \in E}\frac{\left|{ℵ}_{\text{1}}-{ℵ}_{\text{2}}\right|}{\text{2}}+\sqrt[{q}]{\begin{array}{c} \frac{\text{1}}{\text{2}d}\sum_{\rho \in E}({\left|{\left({\text{ɀ}}_{{\text{Ϣ}}_{\text{1}}}(\rho )\right)}^{\text{2}}-{\left({\text{ɀ}}_{{\text{Ϣ}}_{\text{2}}}(\rho )\right)}^{\text{2}}\right|}^{q}+{\left|{\left({\text{ɤ}}_{{\text{Ϣ}}_{\text{1}}}(\rho )\right)}^{\text{2}}-{\left({\text{ɤ}}_{{\text{Ϣ}}_{\text{2}}}(\rho )\right)}^{\text{2}}\right|}^{q}\\ +{\left|{\left(\pi_{{\text{Ϣ}}_{\text{1}}}(\rho )\right)}^{\text{2}}-{\left(\pi_{{\text{Ϣ}}_{\text{2}}}(\rho )\right)}^{\text{2}}\right|}^{q}) \end{array}}\right),$$

where *q = 1, 2*. If *q = 1* and *q = 2*, then distance $H_{\text{3}}^{\text{1}}\left({\text{Ϣ}}_{{ℵ}_{\text{1}}}^{py},\;{\text{Ϣ}}_{{ℵ}_{\text{2}}}^{py}\right)$ and $H_{\text{3}}^{\text{2}}\left({\text{Ϣ}}_{{ℵ}_{\text{1}}}^{py},\;{\text{Ϣ}}_{{ℵ}_{\text{2}}}^{py}\right)$ are known as Szmidt and Kacprzyk’s form of Hamming distance and, Szmidt and Kacprzyk’s form of Euclidean distance for Dia‑*PyFS*s, respectively.

## 7. Applications of Dia‑*PyFS*s in MCDM scenarios via CODAS technique

Input:

Step 1: A team of decision-makers (*DM*rs) should be formed using Dia‑*PyF* data in the Dia‑*PyFS* format for an appropriate number of alternatives and attributes, denoted as *l*_*j*_ (∈ ℕ). Here, Ể = {${\text{Ể}}_{\text{1}}$, ${\text{Ể}}_{\text{2}}$, ..., ${\text{Ể}}_{u}$, ${\text{Ể}}_{l}$} represents a group of experts. The preferences of each experts are evaluated using Dia‑*PyFV*s. Thus, the decision data, expressed as Dia‑*PyFV*s, are arranged in the decision matrix (*M*), including ${\text{Ể}}_{\text{1}}$, ${\text{Ể}}_{\text{2}}$, and ${\text{Ể}}_{\text{3}}$, along with the corresponding weighting vector $\varpi_{\;}$.

Step 2: Standardization of Dia‑*PyF* data inputs:

To achieve optimal and precise results, it is essential to normalize the input data before proceeding with further calculations. As a result, the Dia‑*PyF* analysis can be standardized by


$${\text{𝓁}}_{j}=\left\{ \begin{array}{cc} @lr\left(\left\langle {\text{ɀ}}_{{\text{𝓁}}_{i}},\;{\text{ɤ}}_{{\text{𝓁}}_{i}}\right\rangle \right), & \text{same}\;\text{type}\;\text{input}\;\text{data}\\ \left(\left\langle {\text{ɤ}}_{{\text{𝓁}}_{i}},{\text{ɀ}}_{{\text{𝓁}}_{i}}\right\rangle \right), & \text{different}\;\text{type}\;\text{input}\;\text{data}. \end{array}\right.\;$$

In this case, since the input data for all attributes is identical, there is no need to normalize the data. All alternatives and criteria in our specific problem are of the same nature.

Step 3: Using the Proposition 1, we find Dia‑*PyF* decision matrix (*M*) based on the decision data provided in matrix *M*_*k*_ (*k* = 1, 2, 3) in order to determine the alternatives’ by with help of Proposition 1 overall preference values, *Ą*_*i*_.

Step 4: To complete the data, experts first assign weights to each criterion. This ensures that the final decision reflects the collective input of all health experts.


$$\varpi ={\left(\frac{\text{1}}{r}\sum_{\zeta =\text{1}}^{r}\varpi_{\text{1}}^{\zeta },\frac{\text{1}}{r}\sum_{\zeta =\text{1}}^{r}\varpi_{\text{2}}^{\zeta },\ldots \ldots \ldots ,\frac{\text{1}}{r}\sum_{\zeta =\text{1}}^{r}\varpi_{\text{n}}^{\zeta }\right)}^{T}$$

Step 5: To calculate the weighted Dia‑*PyF* decision matrix, we use following equation for the weight vector $\varpi_{j}$ (*j* = 1, 2, 3, 4, 5) such that:


$$\varpi_{j}{\text{𝓁}}_{ij}=\left\langle {\left(\text{1}-{\left(\text{1}-{\left({\text{ɀ}}_{{\text{𝓁}}_{i}}\right)}^{\text{2}}\right)}^{\varpi_{j}}\right)}^{\frac{\text{1}}{\text{2}}},{\text{ɤ}}_{{\text{𝓁}}_{i}}^{\varpi_{j}};{\left(\text{1}-{\left(\text{1}-{\left({ℵ}_{{\text{𝓁}}_{i}}\right)}^{\text{2}}\right)}^{\varpi_{j}}\right)}^{\frac{\text{1}}{\text{2}}}\right\rangle ,$$

Step 6: Determine the solution that identify the diamond Pythagorean fuzzy ideal solution (FIS) by selecting the maximum value for each criterion across all alternative


$$\text{F}IS=\left\{\left\langle u_{{\text{𝓁}}_{j}},v_{{\text{𝓁}}_{j}};{ℵ}_{{\text{𝓁}}_{j}}\right\rangle ,j=\text{1},\text{2},\text{3},\ldots \ldots \ldots ,\;m\;\right\}=\left\{\left\langle \underset{\text{1}\leq i\leq n}{\text{max}}{\text{ɀ}}_{{\text{𝓁}}_{ij}},\underset{\text{1}\leq i\leq n}{\text{min}}{\text{ɤ}}_{{\text{𝓁}}_{ij}};\underset{\text{1}\leq i\leq n}{\text{max}}{ℵ}_{{\text{𝓁}}_{ij}}\right\rangle ,\;j=\text{1},\text{2},\text{3},\ldots \ldots \ldots \ldots ,\;m\;\right\}.$$

Step 7: For each alternative, compute the Dia‑*PyF* -Hamming and Dia‑*PyF* -Euclidean distance from the *FIS*.


$${\mathrm{\backslash itOmega}}_{i}=H_{\text{2}}^{\text{1}}\left(FIS,\;{\text{Ą}}_{i}\right)=\frac{\text{1}}{\text{2}}\times \left(\frac{\text{1}}{d}\sum_{\rho \in E}\frac{\left|{ℵ}_{\text{1}}-{ℵ}_{\text{2}}\right|}{\text{2}}+\frac{\text{1}}{\text{2}d}\sum_{\rho \in E}\left(\left|{\left({\text{ɀ}}_{{\text{𝓁}}_{ij}}\right)}^{\text{2}}-{\left({\text{ɀ}}_{{\text{𝓁}}_{j}}\right)}^{\text{2}}\right|+\left|{\left({\text{ɤ}}_{{\text{𝓁}}_{ij}}\right)}^{\text{2}}-{\left({\text{ɤ}}_{{\text{𝓁}}_{j}}\right)}^{\text{2}}\right|\right)\right),$$

And


$${\text{Ể}}_{i}=H_{\text{2}}^{\text{2}}\left(FIS,\;{\text{Ą}}_{i}\right)=\frac{\text{1}}{\text{2}}\times \left(\frac{\text{1}}{d}\sum_{\rho \in E}\frac{\left|{ℵ}_{\text{1}}-{ℵ}_{\text{2}}\right|}{\text{2}}+\sqrt[{\;}]{\frac{\text{1}}{\text{2}d}\sum_{\rho \in E}\left({\left|{\left({\text{ɀ}}_{{\text{𝓁}}_{ij}}\right)}^{\text{2}}-{\left({\text{ɀ}}_{{\text{𝓁}}_{j}}\right)}^{\text{2}}\right|}^{\text{2}}+{\left|{\left({\text{ɤ}}_{{\text{𝓁}}_{ij}}\right)}^{\text{2}}-{\left({\text{ɤ}}_{{\text{𝓁}}_{j}}\right)}^{\text{2}}\right|}^{\text{2}}\right)}\right),$$

Or


$${\mathrm{\backslash itOmega}}_{i}=H_{\text{3}}^{\text{1}}\left(FIS,\;{\text{Ą}}_{i}\right)=\frac{\text{1}}{\text{2}}\times \left(\frac{\text{1}}{d}\sum_{\rho \in E}\frac{\left|{ℵ}_{\text{1}}-{ℵ}_{\text{2}}\right|}{\text{2}}+\frac{\text{1}}{\text{2}d}\sum_{\rho \in E}\left(\left|{\left({\text{ɀ}}_{{\text{𝓁}}_{ij}}\right)}^{\text{2}}-{\left({\text{ɀ}}_{{\text{𝓁}}_{j}}\right)}^{\text{2}}\right|+\left|{\left({\text{ɤ}}_{{\text{𝓁}}_{ij}}\right)}^{\text{2}}-{\left({\text{ɤ}}_{{\text{𝓁}}_{j}}\right)}^{\text{2}}\right|+\left|{\left(\pi_{{\text{𝓁}}_{ij}}\right)}^{\text{2}}-{\left(\pi_{{\text{𝓁}}_{j}}\right)}^{\text{2}}\right|\right)\right).$$

and


$${\text{Ể}}_{i}=H_{\text{3}}^{\text{2}}\left(FIS,\;{\text{Ą}}_{i}\right)=\frac{\text{1}}{\text{2}}\times \left(\frac{\text{1}}{d}\sum_{\rho \in E}\frac{\left|{ℵ}_{\text{1}}-{ℵ}_{\text{2}}\right|}{\text{2}}+\sqrt[{\;}]{\frac{\text{1}}{\text{2}d}\sum_{\rho \in E}\left({\left|{\left({\text{ɀ}}_{{\text{𝓁}}_{ij}}\right)}^{\text{2}}-{\left({\text{ɀ}}_{{\text{𝓁}}_{j}}\right)}^{\text{2}}\right|}^{\text{2}}+{\left|{\left({\text{ɤ}}_{{\text{𝓁}}_{ij}}\right)}^{\text{2}}-{\left({\text{ɤ}}_{{\text{𝓁}}_{j}}\right)}^{\text{2}}\right|}^{\text{2}}+{\left|{\left(\pi_{{\text{𝓁}}_{ij}}\right)}^{\text{2}}-{\left(\pi_{{\text{𝓁}}_{j}}\right)}^{\text{2}}\right|}^{\text{2}}\right)}\right).$$

where q=1,2.

Step 8: Construct the relative assessment matrix (RAM) using the calculated values of $H_{\text{2}}^{q}$ and $H_{\text{3}}^{q}$ from the previous step as follows:

$R={\left[R_{ik}\right]}_{m\times m}$,


$$R_{ik}=\left({\text{Ể}}_{i}-{\text{Ể}}_{k}\right)+\left({\mathrm{\backslash itLambda}}_{ik}\left({\text{Ể}}_{i}-{\text{Ể}}_{k}\right)+\left({\mathrm{\backslash itOmega}}_{i}-{\mathrm{\backslash itOmega}}_{k}\right)\right)$$

Where *Λ*_*ik*_ represents a threshold function used to determine whether the Euclidean distances of two alternatives are equivalent. It is defined as follows:


$${\mathrm{\backslash itLambda}}_{ik}=\left\{ \begin{array}{c} @l\text{1}\;if\;\;\left|{\text{Ể}}_{i}-{\text{Ể}}_{k}\right|\leq \gamma \\ \text{0}\;if\;\;\left|{\text{Ể}}_{i}-{\text{Ể}}_{k}\right|>\gamma , \end{array}\right.\;$$

Here, *γ* represents a threshold function used to determine whether the Euclidean distances of two alternatives are equivalent.

Step 9: The attribute with the highest score receives the top rank via RAM and should be selected as the final choice such that:


$$R_{i}=\sum_{k=\text{1}}^{m}R_{ik}.$$

### 7.1. Selection of electric auto rickshaw via Dia‑*PyFSs*

This section presents a practical case study focused on evaluating and selecting a location for an electric auto rickshaw shredding facility in the Asian countries. The developed Dia‑*PyF* CODAS method is applied to determine the optimal facility location from a set of four possible alternatives, considering a range of conflicting quantitative and qualitative evaluation criteria.

An electric auto rickshaw, or e-rickshaw, is a three-wheeled electric vehicle well-suited for short-range transportation within urban and suburban areas. It presents an environmentally friendly alternative to traditional gasoline or diesel-powered rickshaws by minimizing both air pollution and noise in crowded cities. Powered by rechargeable batteries-typically lithium-ion or lead-acid—these vehicles generally have a range of 50–120 kilometers on a full charge, depending on the model and battery size. Choosing an electric auto-rickshaw offers several environmental and economic advantages. As they produce zero tailpipe emissions, electric rickshaws help improve air quality, especially in high-density areas. They are also quieter than their fuel-powered counterparts, making them a less intrusive option for urban transportation. With fewer moving components, electric auto-rickshaws incur lower maintenance expenses over time, and the cost of recharging the battery is often significantly lower than refueling with gasoline or diesel. Electric auto-rickshaws are generally designed for low to moderate speed, typically achieving speeds between 25 and 35 km/h, which is adequate for short, frequent city trips. With seating for two to four passengers, depending on the model, they are efficient for local commuting. When selecting an electric auto-rickshaw, potential buyers should consider charging infrastructure, as it affects convenience and operational range. In some areas, dedicated charging stations are available, while in others, e-rickshaws can be recharged at home overnight or utilize battery-swapping systems at specific locations. Opting for an electric auto-rickshaw provides several benefits, including reduced environmental impact, minimal noise, and cost savings for both drivers and riders. Many governments support electric vehicle use through incentives, subsidies, or tax breaks, promoting them as part of clean energy and sustainable mobility initiatives. However, there are factors to consider before selecting an electric auto-rickshaw. Limited battery capacity and charging durations may constrain travel range, and charging facilities may not be evenly distributed across regions. Additionally, these vehicles are usually lightweight and best suited to paved roads, which can limit their performance on rough surfaces or with heavier loads. Electric auto-rickshaws have gained significant traction in South Asian nations like Pakistan, Sri lanka, Bangladesh, Nepal, and India, where they serve as an affordable and eco-friendly transportation option. In densely populated urban centers, electric rickshaws meet the demand for economical, low-emission transit, making them an effective solution for sustainable urban transportation.

Four three-wheeled electric auto-rickshaws (**Ą**_**1**_, **Ą**_**2**_, **Ą**_**3**_, and **Ą**_**4**_) were evaluated by three experts (DM_1_, DM_2_, and DM_3_) based on five criteria (${\hat{\text{C}}}_{\text{1}}$, ${\hat{\text{C}}}_{\text{2}}$, ${\hat{\text{C}}}_{\text{3}}$, ${\hat{\text{C}}}_{\text{4}}$, and ${\hat{\text{C}}}_{\text{5}}$) such that:

Standard Passenger Electric Auto-rickshaws $\left({\text{Ą}}_{1}\right)$: These are the most common types of electric auto-rickshaws, designed for short-distance travel in urban and semi-urban areas. They typically carry 2–4 passengers and have a compact body that allows them to navigate through narrow lanes and crowded streets. These vehicles have an open body design, with no doors or windows, offering a more affordable option. They are commonly used in areas with warm climates and are suitable for short-distance travel in cities and towns.

Deluxe or Premium Passenger Electric Auto-rickshaws $\left({\text{Ą}}_{2}\right)$: These vehicles are designed to provide more comfort and amenities, such as cushioned seating, better suspension, air conditioning, and sometimes entertainment systems. They may also have more spacious interiors and are often used for premium services or tourist transport. These auto-rickshaws feature closed cabins, offering more protection from weather elements such as rain or extreme heat. The enclosed body provides a more comfortable ride, making them suitable for urban areas with variable weather conditions.

Convertible Passenger Electric Auto-rickshaws $\left({\text{Ą}}_{3}\right)$: These vehicles allow for flexibility in usage. The rear section can be adjusted or converted to accommodate more passengers or cargo, depending on demand. This type is useful for operators who wish to switch between passenger transport and cargo delivery without requiring multiple vehicles.

Solar-Powered Passenger Electric Auto-rickshaws $\left({\text{Ą}}_{4}\right)$: These models incorporate solar panels on the roof, which help recharge the battery during the day, extending the vehicle’s range. Solar-powered e-rickshaws are particularly useful in regions with abundant sunlight and can reduce operating costs by using renewable energy.

**Fig 5:**
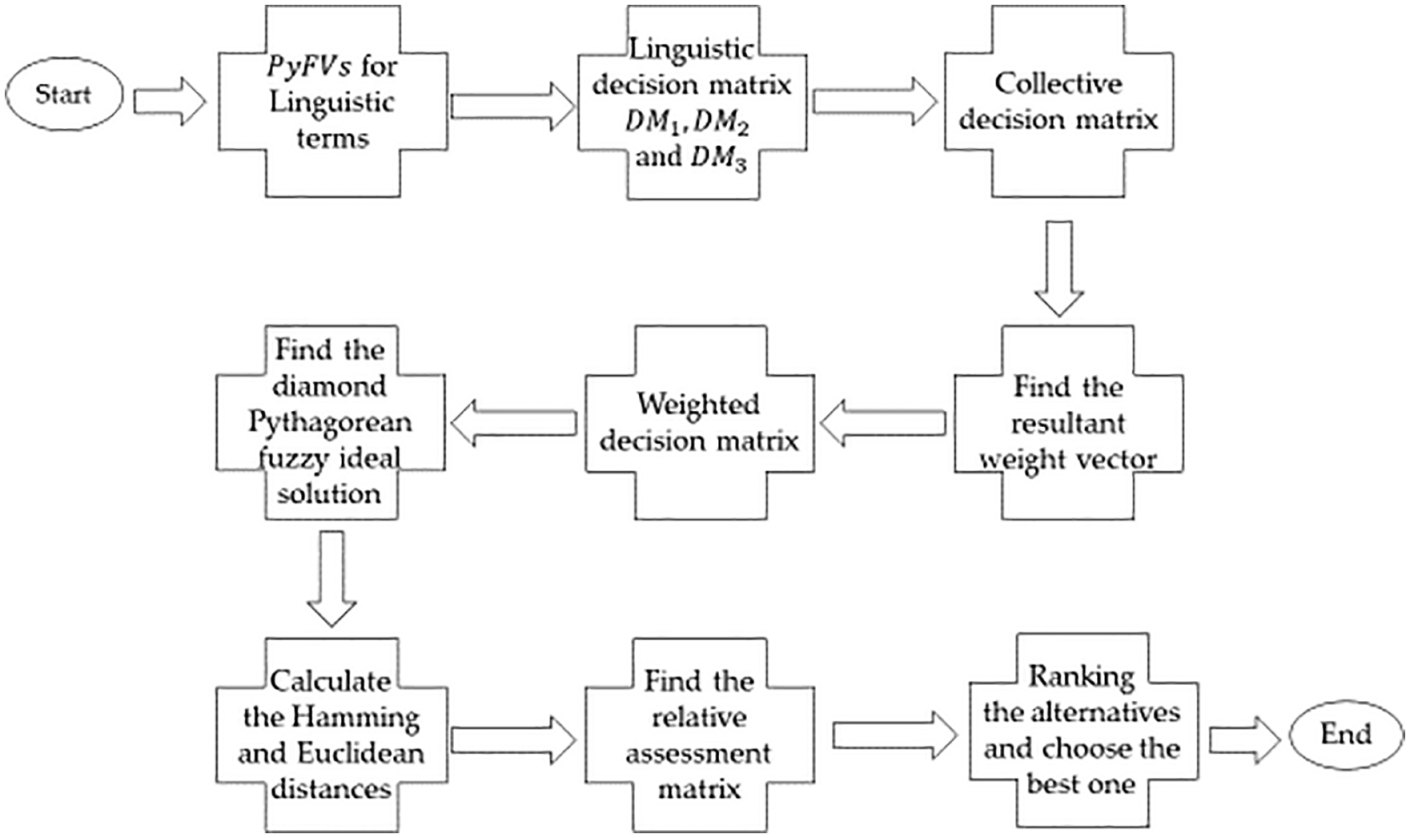
Flow chart of the proposed algorithm.

Following are the criteria that will help to find best alternative such that:

**Battery-Powered** (${\hat{𝐂}}_{1}$): Electric auto-rickshaws are powered by rechargeable batteries, commonly lithium-ion or lead-acid batteries. Battery capacity varies, with a typical range of around 50–120 kilometers per full charge, depending on the model and battery type.
**Environmentally Friendly** (${\hat{𝐂}}_{2}$): E-rickshaws produce zero tailpipe emissions, making them an eco-friendly solution in cities striving to reduce pollution levels. With electricity as their fuel source, they contribute less to air pollution and are quieter than traditional fuel-powered auto-rickshaws.
**Speed and Performance** (${\hat{𝐂}}_{3}$): Typically, e-rickshaws are designed for low-speed travel, with maximum speeds ranging from 25–35 km/h (15–22 mph). They are optimized for short, intra-city routes, carrying 2–4 passengers over relatively short distances.
**Charging Infrastructure** (${\hat{𝐂}}_{4}$): The expansion of charging stations is critical for e-rickshaws to operate efficiently in large numbers. In many regions, charging infrastructure is being developed specifically for these vehicles. Some e-rickshaws can be charged overnight at home, while others may use swappable battery systems at designated stations for convenience.
**Load Capacity** (${\hat{𝐂}}_{5}$): Electric auto-rickshaws are generally designed for lightweight transportation and may not be as robust as traditional models when carrying heavier loads or navigating rough terrains. The suggested algorithm's geometrical interpretation is presented in the form of Fig 5.

### 7.2. Using Algorithm 1 to solve

Step 1: We apply this algorithm to process the input data, where three medical professionals are tasked with evaluating four emergency options, *Ą*_*i*_ (i = 1, 2, 3, 4), in relation to five criteria ${\hat{\text{C}}}_{i}$ (i = 1, 2, 3, 4, 5). For the construction of the decision matrices for the three experts, *DM*_*ζ*_ (ζ = 1, 2, 3), and the criteria ${\hat{\text{C}}}_{i}$, the *PyFVs* for Linguistic terms are given in Table 1.

**Table 1:** *PyFVs* for Linguistic terms.

Linguistic terms	Acronym	*PyFVs*
Extremely good	EG	⟨0.9,0.1⟩
Very very good	VVG	⟨0.7,0.4⟩
Very good	VG	⟨0.5,0.4⟩
good	G	⟨0.6,0.6⟩
Medium good	MG	⟨0.4,0.6⟩
Medium	M	⟨0.5,0.5⟩
Medium bad	MB	⟨0.5,0.6⟩
Bad	B	⟨0.4,0.7⟩
Very bad	VB	⟨0.4,0.8⟩
Very very bad	VVB	⟨0.3,0.9⟩
Extremely bad	EB	⟨0.2,0.9⟩

Step 2: The expert assessments for decision-making have been collected and recorded using *PyF* phrases, as shown in Tables 2–. The *PyF*DM, outlined in Tables 5– are derived from Tables 2–. respectively, using the *PyFVs* defined in Table 1.

**Table 2:** Linguistic decision matrix *DM*_*1*_ via *PyFV*s.

	${\hat{𝐂}}_{1}$	${\hat{𝐂}}_{2}$	${\hat{𝐂}}_{3}$	${\hat{𝐂}}_{4}$	${\hat{𝐂}}_{5}$
*Ą*_*1*_	G	M	B	VVB	MB
*Ą*_*2*_	MG	EB	VVG	VG	VB
*Ą*_*3*_	VVG	G	VVB	EB	MG
*Ą*_*4*_	VVB	B	VG	VB	G

**Table 3:** Linguistic decision matrix *DM*_2_ via *PyFV*s.

	${\hat{𝐂}}_{1}$	${\hat{𝐂}}_{2}$	${\hat{𝐂}}_{3}$	${\hat{𝐂}}_{4}$	${\hat{𝐂}}_{5}$
*Ą*_*1*_	MB	B	M	G	VVB
*Ą*_*2*_	VB	VVG	EB	MG	VG
*Ą*_*3*_	MG	VVB	G	VVG	EB
*Ą*_*4*_	M	VG	B	VVB	VB

**Table 4:** Linguistic decision matrix *DM*_*3*_ via *PyFV*s.

	${\hat{𝐂}}_{1}$	${\hat{𝐂}}_{2}$	${\hat{𝐂}}_{3}$	${\hat{𝐂}}_{4}$	${\hat{𝐂}}_{5}$
*Ą*_*1*_	M	VVG	MB	G	B
*Ą*_*2*_	EG	VG	VB	MG	VVG
*Ą*_*3*_	G	EB	MG	VVG	VVB
*Ą*_*4*_	B	VB	M	VVB	VG

**Table 5:** Decision matrix *DM*_*1*_ via *PyFV*s.

	${\hat{𝐂}}_{1}$	${\hat{𝐂}}_{2}$	${\hat{𝐂}}_{3}$	${\hat{𝐂}}_{4}$	${\hat{𝐂}}_{5}$
*Ą*_*1*_	⟨0.6,0.6⟩	⟨0.5,0.5⟩	⟨0.4,0.7⟩	⟨0.3,0.9⟩	⟨0.5,0.6⟩
*Ą*_*2*_	⟨0.4,0.6⟩	⟨0.2,0.9⟩	⟨0.7,0.4⟩	⟨0.5,0.4⟩	⟨0.4,0.8⟩
*Ą*_*3*_	⟨0.7,0.4⟩	⟨0.6,0.6⟩	⟨0.3,0.9⟩	⟨0.2,0.9⟩	⟨0.4,0.6⟩
*Ą*_*4*_	⟨0.3,0.9⟩	⟨0.5,0.6⟩	⟨0.5,0.4⟩	⟨0.4,0.8⟩	⟨0.6,0.6⟩

**Table 6:** Decision matrix *DM*_*2*_ via *PyFV*s.

	${\hat{𝐂}}_{1}$	${\hat{𝐂}}_{2}$	${\hat{𝐂}}_{3}$	${\hat{𝐂}}_{4}$	${\hat{𝐂}}_{5}$
*Ą*_*1*_	⟨0.5,0.6⟩	⟨0.4,0.7⟩	⟨0.5,0.5⟩	⟨0.6,0.6⟩	⟨0.3,0.9⟩
*Ą*_*2*_	⟨0.4,0.8⟩	⟨0.7,0.4⟩	⟨0.2,0.9⟩	⟨0.4,0.6⟩	⟨0.5,0.4⟩
*Ą*_*3*_	⟨0.4,0.6⟩	⟨0.3,0.9⟩	⟨0.6,0.6⟩	⟨0.7,0.4⟩	⟨0.2,0.9⟩
*Ą*_*4*_	⟨0.5,0.5⟩	⟨0.5,0.4⟩	⟨0.4,0.7⟩	⟨0.3,0.9⟩	⟨0.4,0.8⟩

**Table 7:** Decision matrix *DM*_*3*_ via *PyFV*s.

	${\hat{\text{C}}}_{1}$	${\hat{𝐂}}_{2}$	${\hat{𝐂}}_{3}$	${\hat{𝐂}}_{4}$	${\hat{𝐂}}_{5}$
*Ą*_*1*_	⟨0.5,0.5⟩	⟨0.7,0.4⟩	⟨0.5,0.6⟩	⟨0.6,0.6⟩	⟨0.4,0.7⟩
*Ą*_*2*_	⟨0.9,0.1⟩	⟨0.5,0.4⟩	⟨0.4,0.8⟩	⟨0.4,0.6⟩	⟨0.7,0.4⟩
*Ą*_*3*_	⟨0.6,0.6⟩	⟨0.2,0.9⟩	⟨0.4,0.6⟩	⟨0.7,0.4⟩	⟨0.3,0.9⟩
*Ą*_*4*_	⟨0.4,0.7⟩	⟨0.4,0.8⟩	⟨0.5,0.5⟩	⟨0.3,0.9⟩	⟨0.5,0.4⟩

Step 3: Next, we calculate the combined Dia‑*PyF* information using the Proposition 1, as detailed in Table 8.

**Table 8:** Collective decision matrix (*DM*) via Dia‑*PyFV*s.

	${\hat{𝐂}}_{1}$	${\hat{𝐂}}_{2}$	${\hat{𝐂}}_{3}$	${\hat{𝐂}}_{4}$	${\hat{𝐂}}_{5}$
*Ą*_*1*_	⟨0.29,0.32;0.59⟩	⟨0.30,0.30;0.50⟩	⟨0.22,0.37;0.51⟩	⟨0.27,0.51;0.42⟩	⟨0.17,0.55;0.48⟩
*Ą*_*2*_	⟨0.38,0.34;0.76⟩	⟨0.26,0.38;0.58⟩	⟨0.23,0.54;0.61⟩	⟨0.19,0.29;0.52⟩	⟨0.30,0.32;0.58⟩
*Ą*_*3*_	⟨0.34,0.29;0.57⟩	⟨0.16,0.66;0.50⟩	⟨0.20,0.51;0.49⟩	⟨0.34,0.38;0.66⟩	⟨0.10,0.66;0.44⟩
*Ą*_*4*_	⟨0.17,0.52;0.52⟩	⟨0.22,0.39;0.59⟩	⟨0.22,0.30;0.58⟩	⟨0.11,0.75;0.33⟩	⟨0.26,0.39;0.56⟩

Step 4: The five weight vectors for the Dia‑*PyF* data are presented as follows:


$$w_{\text{1}}{=(\text{0}.\text{5},\;\text{0}.\text{1}\text{5},\;\text{0}.\text{1},\;\text{0}.\text{1}\text{5},\;\text{0}.\text{1})}^{T}$$

$$w_{\text{2}}{=(\text{0}.\text{4},\;\text{0}.\text{2},\;\text{0}.\text{1},\;\text{0}.\text{2},\;\text{0}.\text{1})}^{T}$$

$$w_{\text{3}}{=(\text{0}.\text{3},\;\text{0}.\text{3},\;\text{0}.\text{2},\;\text{0}.\text{1},\;\text{0}.\text{1})}^{T}$$

$$w_{\text{4}}{=(\text{0}.\text{3}\text{5},\;\text{0}.\text{2}\text{5},\text{0}.\text{1}\text{5},\;\text{0}.\text{1}\text{5},\;\text{0}.\text{1})}^{T}$$

$$w_{\text{5}}{=(\text{0}.\text{2}\text{5},\;\text{0}.\text{3}\text{5},\text{0}.\text{2},\;\text{0}.\text{1},\;\text{0}.\text{1})}^{T}$$

The weights are calculated using the formula given in Step 4, resulting in the final weight vector: $\varpi {=(\text{0}.\text{3}\text{6},\;\text{0}.\text{2}\text{5},\text{0}.\text{1}\text{5},\;\text{0}.\text{1}\text{4},\;\text{0}.\text{1})}^{T}$. This vector clearly satisfies the condition $\sum_{j=\text{1}}^{\text{5}}\varpi_{j}=\text{1}$.

Step 5: We determine the Dia‑*PyFDM* using the weight vector ${\varpi =(\text{0}.\text{3}\text{6},\;\text{0}.\text{2}\text{5},\text{0}.\text{1}\text{5},\;\text{0}.\text{1}\text{4},\;\text{0}.\text{1})}^{T}$. Consequently, we obtain the weighted Dia‑*PyFDM* decision matrices, as shown in Table 9.

**Table 9:** Weighted decision matrix (*DM*) via Dia‑*PyFV*s.

	${\hat{\text{C}}}_{1}$	${\hat{\text{C}}}_{2}$	${\hat{\text{C}}}_{3}$	${\hat{\text{C}}}_{4}$	${\hat{\text{C}}}_{5}$
*Ą*_*1*_	⟨0.17,0.67;0.38⟩	⟨0.15,0.74;0.26⟩	⟨0.09,0.86;0.21⟩	⟨0.10,0.91;0.16⟩	⟨0.05,0.94;0.16⟩
*Ą*_*2*_	⟨0.23,0.68;0.52⟩	⟨0.13,0.78;0.31⟩	⟨0.09,0.91;0.26⟩	⟨0.07,0.84;0.21⟩	⟨0.10,0.89;0.20⟩
*Ą*_*3*_	⟨0.21,0.64;0.36⟩	⟨0.08,0.90;0.26⟩	⟨0.08,0.90;0.20⟩	⟨0.13,0.87;0.28⟩	⟨0.03,0.96;0.15⟩
*Ą*_*4*_	⟨0.10,0.79;0.33⟩	⟨0.11,0.79;0.32⟩	⟨0.09,0.83;0.24⟩	⟨0.04,0.96;0.13⟩	⟨0.08,0.91;0.19⟩

Step 6: For diamond Pythagorean fuzzy ideal solution, we have


FIS={⟨0.23, 0.64;52⟩, ⟨0.15, 0.74;32⟩, ⟨0.09, 0.83;26⟩,⟨0.13, 0.84;28⟩,⟨0.10, 0.89;20⟩}.

Step 7: Next, we calculate the Hamming and Euclidean distances of each alternative *Ą*_*i*_ from *FIS*. We represent these distances as $H_{\text{2}}^{\text{1}}$ and $H_{\text{2}}^{\text{2}}$, with the results as follows:


$$H_{\text{2}}^{\text{1}}\left({\text{Ą}}_{1}\right)=\text{0}.\text{0}\text{4},\;H_{\text{2}}^{\text{1}}\left({\text{Ą}}_{\text{2}}\right)=\text{0}.\text{0}\text{2},\;H_{\text{2}}^{\text{1}}\left({\text{Ą}}_{\text{3}}\right)=\text{0}.\text{0}\text{5},\;H_{\text{2}}^{\text{1}}\left({\text{Ą}}_{\text{4}}\right)=\text{0}.\text{0}\text{5},$$

and


$$H_{\text{2}}^{\text{2}}({\text{Ą}}_{1})=\text{0}.\text{2}\text{1},\;H_{\text{2}}^{\text{2}}\left({\text{Ą}}_{\text{2}}\right)=\text{0}.\text{1}\text{7},\;H_{\text{2}}^{\text{2}}\left({\text{Ą}}_{\text{3}}\right)=\text{0}.\text{2}\text{9},\;H_{\text{2}}^{\text{2}}\left({\text{Ą}}_{\text{4}}\right)=\text{0}.\text{2}\text{4}.$$

Step 8: The relative assessment matrix is derived from the above data using equations in previous Step 8. Here, threshold parameter is set at a value of 0.15.


$$(\begin{array}{cccc} \text{0} & \text{0}.\text{0}\text{4}\text{0} & -\text{0}.\text{0}\text{7}\text{9} & -\text{0}.\text{0}\text{3}\text{9}\text{9}\\ -\text{0}.\text{0}\text{4}\text{0} & \text{0} & -\text{0}.\text{1}\text{1}\text{9} & -\text{0}.\text{0}\text{6}\text{9}\text{7}\\ \text{0}.\text{0}\text{8}\text{0} & \text{0}.\text{1}\text{2}\text{1} & \text{0} & \text{0}.\text{0}\text{5}\text{0}\\ \text{0}.\text{3}\text{0}\text{0} & \text{0}.\text{0}\text{7}\text{0} & -\text{0}.\text{0}\text{5}\text{0} & \text{0} \end{array}).$$

Step 9: The score of four potential options are determine by summing of each of the row such that *R*_*1*_* = −0.079*, *R*_*2*_* = −0.309*, *R*_*3*_* = 251*, and *R*_*4*_* = .320* and then are prioritized according to the decreasing values of their assessment scores. The order of ranking is as follows: Ą_4_ ≻ Ą_3_ ≻ Ą_1_ ≻ Ą_2_. Thus, based on the diamond Pythagorean fuzzy CODAS approach, “solar-powered passenger electric auto-rickshaws” is identified as the most suitable ride for the people in the Asian country.

As this is pioneering research, there are currently no alternative solutions available for direct comparison using a pure Dia‑*PyF* framework. Therefore, this section contrasts the proposed methodologies with established *MCDM* approaches, emphasizing their application in a *PyF* environment. Since both Dia‑*PyF* expand upon traditional *PyFs*, we can obtain a *PyF* from any Dia‑*PyF* by setting **ℵ* = 0* across all elements. Applying this approach to the data from our case study yields results also summarized in Table 10. It is observed that the ranking of alternatives changes and is also influenced by the specific *MCDM* method used. However, our methodologies offer the advantage of fully utilizing the information provided by experts, as they operate within the broader Dia‑*PyF* environment. Notably, the Dia‑*PyF* CODAS method is enhanced through the use of empirically effective Hamming and Euclidean distance measures.

**Table 10:** Analyzing the intended work in comparison to the classical work.

Authors	ℵ	Methods	Applicable or not applicable
Khan et al. [24]	No	VIKOR method	Not calculable
Akram et al. [25]	No	ELECTRE-II method	Not calculable
Peng and Yang [26]	No	MABAC method	Not calculable
Khan et al. [27]	No	*PyF*DWA operator	Not calculable
Ashraf et al. [28]	No	ST-PFWA operator	Not calculable
Garg [29]	No	*CPyF*EWA operator	Not calculable
Zhang and Xu [30]	No	TOPSIS method	Not calculable
Bozyigit et al. [24]	No	*PyF*WGA operator	Not calculable
Proposed Method	Yes	CODAS method	Ą_4_ ≻ Ą_3_ ≻ Ą_1_ ≻ Ą_2_

## 8. Conclusion

This study introduces the Dia‑*PyFS*, focusing on its diamond structure as a primary feature. The Dia‑*PyFS* forms a diamond with a norm capped at two and a center defined by validity and non-validity values. By encapsulating data within a diamond structure, the two-dimensional Dia‑*PyFS* can effectively manage high-level inaccurate data. The properties of algebraic operations within Dia‑*PyFSs* are explored. Using the *PyF* t-norm and t-conorm, weighted and geometric operations for Dia‑*PyFS* are defined within this framework. Additionally, DM via *PyFS* and DM via Dia‑*PyFS* are examined to measure the gap between the aggregated value and the optimal solution. The proposed aggregation operators and *CODAS* methods provide tools for selecting an electric autorickshaw based on multiple criteria. The suggested operations and DMs are guaranteed to be reliable through comparison and visualization.

In *PyFS* and *IVPyFS* frameworks, input data consist of a point and an interval along a single dimension, whereas, in the Dia‑*PyF* framework, the input data form a two-dimensional diamond. This diamond approach in Dia‑*PyFSs* provides a broader scope for handling uncertainty compared to *PyFS* and *IVPyFS*, making it particularly useful for applications requiring high accuracy in managing imprecision. For instance, Dia‑*PyFSs* offer enhanced effectiveness in fields such as machine learning, deep learning, pattern recognition, and areas like medical diagnostics. Future research will primarily focus on exploring alternative t-norm and t-conorm operators for aggregating Dia‑*PyFSs*. Furthermore, transforming *PyFS* clusters into Dia‑*PyFSs* may allow for the definition and application of diverse distance and similarity metrics between clusters in fields like computer image analysis and medical treatment. Additionally, the diamond structure could be applied to specific values, such as orthopair fuzzy sets, and generalized fuzzy sets, such as quasi rung orthopair fuzzy sets and picture fuzzy sets. Aggregation operations and distance measures of these developed sets will also be utilized in collaborative decision-making processes.

## References

[1] Zadeh LA. Fuzzy sets. Information and Control. 1965;8(3):338–53. doi: 10.1016/s0019-9958(65)90241-x

[2] Atanassov K, Gargov G. Interval valued intuitionistic fuzzy sets. Fuzzy Sets and Systems. 1989;31:343–9.

[3] Huang Z, Li K, Jiang Y, Jia Z, Lv L, Ma Y. Graph Relearn Network: Reducing performance variance and improving prediction accuracy of graph neural networks. Knowledge-Based Systems. 2024;301:112311. doi: 10.1016/j.knosys.2024.112311

[4] Ling W, Jing C, Wan J, Mao A, Xiao Q, Guan J, et al. Design and construction of the near-earth space plasma simulation system of the Space Plasma Environment Research Facility. J Plasma Phys. 2024;90(1). doi: 10.1017/s0022377823001460

[5] Xie J, Wen M, Ding P, Tu Y, Wu D, Liu K, et al. Interfacial flow contact resistance effect for thermal consolidation of layered viscoelastic saturated soils with semi‐permeable boundaries. Num Anal Meth Geomechanics. 2024;48(15):3640–79. doi: 10.1002/nag.3805

[6] Wang J, Ji J, Jiang Z, Sun L. Traffic Flow Prediction Based on Spatiotemporal Potential Energy Fields. IEEE Trans Knowl Data Eng. 2023;35(9):9073–87. doi: 10.1109/tkde.2022.3221183

[7] Zhang D, Pee LG, Pan SL, Wang J. Information practices in data analytics for supporting public health surveillance. Asso for Info Science & Tech. 2023;75(1):79–93. doi: 10.1002/asi.24841

[8] Zhao Z, Zhang H, Shiau J, Du W, Ke L, Wu F, et al. Failure envelopes of rigid tripod pile foundation under combined vertical-horizontal-moment loadings in clay. Applied Ocean Research. 2024;150:104131. doi: 10.1016/j.apor.2024.104131

[9] Gao Y, Liu Q, Yang Y, Wang K. Latent representation discretization for unsupervised text style generation. Information Processing & Management. 2024;61(3):103643. doi: 10.1016/j.ipm.2024.103643

[10] Zhang Y. Multi-slicing strategy for the three-dimensional discontinuity layout optimization (3D DLO). Int J Numer Anal Methods Geomech. 2017;41(4):488–507. doi: 10.1002/nag.2566 28303076 PMC5324696

[11] Yager RR. Pythagorean fuzzy subsets. In: Proceedings of the 2013 Joint IFSA World Congress and NAFIPS Annual Meeting. 2013. 57–61.

[12] Araral E. Why do cities adopt smart technologies? Contingency theory and evidence from the United States. Cities. 2020;106:102873. doi: 10.1016/j.cities.2020.102873

[13] Atanassov KT. Circular intuitionistic fuzzy sets. IFS. 2020;39(5):5981–6. doi: 10.3233/jifs-189072

[14] Fan J, Pan Y, Wang H, Song F. Efficient reverse osmosis-based desalination using functionalized graphene oxide nanopores. Appl Surf Sci. 2024;674:160937.

[15] Huang B, Kang F, Li X, Zhu S. Underwater dam crack image generation based on unsupervised image-to-image translation. Automation in Construction. 2024;163:105430. doi: 10.1016/j.autcon.2024.105430

[16] Wu Y, Kang F, Zhang Y, Li X, Li H. Structural identification of concrete dams with ambient vibration based on surrogate-assisted multi-objective salp swarm algorithm. Structures. 2024;60:105956. doi: 10.1016/j.istruc.2024.105956

[17] Ren H, Xia X, Sun Y, Zhai Y, Zhang Z, Wu J, et al. Electrolyte engineering for the mass exfoliation of graphene oxide across wide oxidation degrees. J Mater Chem A. 2024;12(35):23416–24. doi: 10.1039/d4ta02654c

[18] Wang C, Yang L, Hu M, Wang Y, Zhao Z. On-demand airport slot management: tree-structured capacity profile and coadapted fire-break setting and slot allocation. Transportmetrica A: Transport Science. 2024;:1–35.

[19] Lu Y, Guo Z, Zhang M, Zhang M, Jiang X, Wang X. Flow-heat coupling analysis of the 1/4 symmetrical CAP1400 nuclear island loop based on the source term approach. Annals of Nuclear Energy. 2025;211:110926. doi: 10.1016/j.anucene.2024.110926

[20] Wu Z, Ma C, Zhang L, Gui H, Liu J, Liu Z. Predicting and compensating for small-sample thermal information data in precision machine tools: A spatial-temporal interactive integration network and digital twin system approach. Applied Soft Computing. 2024;161:111760. doi: 10.1016/j.asoc.2024.111760

[21] Lu Y, Qin L, Mao Y, Lnong X, Wei Q, Su J, et al. Antibacterial activity of a polysaccharide isolated from litchi (Litchi chinensis Sonn.) pericarp against Staphylococcus aureus and the mechanism investigation. Int J Biol Macromol. 2024;279(Pt 1):134788. doi: 10.1016/j.ijbiomac.2024.134788 39173786

[22] Bao X, Li J, Shen J, Chen X, Zhang C, Cui H. Comprehensive multivariate joint distribution model for marine soft soil based on the vine copula. Computers and Geotechnics. 2025;177:106814. doi: 10.1016/j.compgeo.2024.106814

[23] Zhang X, Usman M, Irshad A ur R, Rashid M, Khattak A. Investigating Spatial Effects through Machine Learning and Leveraging Explainable AI for Child Malnutrition in Pakistan. IJGI. 2024;13(9):330. doi: 10.3390/ijgi13090330

[24] Khan MJ, Ali MI, Kumam P, Kumam W, Aslam M, Alcantud JCR. Improved generalized dissimilarity measure‐based VIKOR method for Pythagorean fuzzy sets. Int J of Intelligent Sys. 2021;37(3):1807–45. doi: 10.1002/int.22757

[25] Akram M, Ilyas F, Garg H. ELECTRE-II method for group decision-making in Pythagorean fuzzy environment. Appl Intell. 2021;51(12):8701–19. doi: 10.1007/s10489-021-02200-0

[26] Peng X, Yang Y. Pythagorean Fuzzy Choquet Integral Based MABAC Method for Multiple Attribute Group Decision Making. Int J Intell Syst. 2016;31(10):989–1020. doi: 10.1002/int.21814

[27] Khan AA, Ashraf S, Abdullah S, Qiyas M, Luo J, Khan SU. Pythagorean Fuzzy Dombi Aggregation Operators and Their Application in Decision Support System. Symmetry. 2019;11(3):383. doi: 10.3390/sym11030383

[28] Ashraf S, Abdullah S, Khan S. Fuzzy decision support modeling for internet finance soft power evaluation based on sine trigonometric Pythagorean fuzzy information. J Ambient Intell Human Comput. 2020;12(2):3101–19. doi: 10.1007/s12652-020-02471-4

[29] Garg H. A New Generalized Pythagorean Fuzzy Information Aggregation Using Einstein Operations and Its Application to Decision Making. Int J Intell Syst. 2016;31(9):886–920. doi: 10.1002/int.21809

[30] Zhang X, Xu Z. Extension of TOPSIS to Multiple Criteria Decision Making with Pythagorean Fuzzy Sets. Int J Intell Syst. 2014;29(12):1061–78. doi: 10.1002/int.21676

[31] Xiao F, Ding W. Divergence measure of Pythagorean fuzzy sets and its application in medical diagnosis. Applied Soft Computing. 2019;79:254–67. doi: 10.1016/j.asoc.2019.03.043

[32] Bozyigit MC, Olgun M, Ünver M. Circular Pythagorean Fuzzy Sets and Applications to Multi-Criteria Decision Making. Informatica. 2023;34(4):713–42. doi: 10.15388/23-infor529

[33] Jumaah FM, Zadain AA, Zaidan BB, Hamzah AK, Bahbibi R. Decision-making solution based multi-measurement design parameter for optimization of GPS receiver tracking channels in static and dynamic real-time positioning multipath environment. Measurement. 2018;118:83–95. doi: 10.1016/j.measurement.2018.01.011

[34] Moslem S, Solieman H, Oubahman L, Duleba S, Senapati T, Pilla F. Assessing Public Transport Supply Quality: A Comparative Analysis of Analytical Network Process and Analytical Hierarchy Process. J Soft Comput Decis Anal. 2023;1(1):124–38. doi: 10.31181/jscda11202311

[35] Kolahi-Randji S, Yousefi Nejad Attari M, Ala A. Enhancement the Performance of Multi-Level and Multi-Commodity in Supply Chain: A Simulation Approach. J Soft Comput Decis Anal. 2023;1(1):18–38. doi: 10.31181/jscda1120232

[36] Fazlollahtabar H, Kazemitash N. Green supplier selection based on the information system performance evaluation using the integrated best-worst method. FU Mech Eng. 2021;19(3):345. doi: 10.22190/fume201125029f

[37] Karamaşa Ç, Karabasevic D, Stanujkic D, Kookhdan AR, Mishra AR, Ertürk M. An extended single-valued neutrosophic ahp and multimoora method to evaluate the optimal training aircraft for flight training organizations. FU Mech Eng. 2021;19(3):555. doi: 10.22190/fume210521059k

[38] Albahri OS, AlSattar HA, Garfan S, Qahtan S, Zaidan AA, Ahmaro IYY, et al. Combination of Fuzzy-Weighted Zero-Inconsistency and Fuzzy Decision by Opinion Score Methods in Pythagorean m-Polar Fuzzy Environment: A Case Study of Sign Language Recognition Systems. Int J Info Tech Dec Mak. 2022;22(04):1341–69. doi: 10.1142/s0219622022500183

[39] Salih MM, Zaidan BB, Zaidan AA. Fuzzy decision by opinion score method. Applied Soft Computing. 2020;96:106595. doi: 10.1016/j.asoc.2020.106595

[40] Baqer MJ, AlSattar HA, Qahtan S, Zaidan AA, Izhar MAM, Abbas IT. A Decision Modeling Approach for Data Acquisition Systems of the Vehicle Industry Based on Interval-Valued Linear Diophantine Fuzzy Set. Int J Info Tech Dec Mak. 2023;24(01):89–168. doi: 10.1142/s0219622023500487

[41] Qahtan S, Yatim K, Zulzalil H, Osman MH, Zaidan AA, Alsattar HA. Review of healthcare industry 4.0 application-based blockchain in terms of security and privacy development attributes: Comprehensive taxonomy, open issues and challenges and recommended solution. Journal of Network and Computer Applications. 2023;209:103529. doi: 10.1016/j.jnca.2022.103529

[42] Sato Y, Tan KH. Inconsistency indices in pairwise comparisons: an improvement of the Consistency Index. Ann Oper Res. 2022;326(2):809–30. doi: 10.1007/s10479-021-04431-3

[43] Qahtan S, Alsattar HA, Zaidan AA, Deveci M, Pamucar D, Delen D. Performance assessment of sustainable transportation in the shipping industry using a q-rung orthopair fuzzy rough sets-based decision making methodology. Expert Systems with Applications. 2023;223:119958. doi: 10.1016/j.eswa.2023.119958

[44] Qahtan S, Alsattar HA, Zaidan AA, Deveci M, Pamucar D, Delen D, et al. Evaluation of agriculture-food 4.0 supply chain approaches using Fermatean probabilistic hesitant-fuzzy sets based decision making model. Applied Soft Computing. 2023;138:110170. doi: 10.1016/j.asoc.2023.110170

[45] Ibrahim HA, Zaidan AA, Qahtan S, Zaidan BB. Sustainability assessment of palm oil industry 4.0 technologies in a circular economy applications based on interval-valued Pythagorean fuzzy rough set-FWZIC and EDAS methods. Applied Soft Computing. 2023;136:110073. doi: 10.1016/j.asoc.2023.110073

[46] Ismael SF, Alias AH, Zaidan AA, Zaidan BB, Alsattar HA, Qahtan S, et al. Toward Sustainable Transportation: A Pavement Strategy Selection Based on the Extension of Dual-Hesitant Fuzzy Multicriteria Decision-Making Methods. IEEE Trans Fuzzy Syst. 2023;31(2):380–93. doi: 10.1109/tfuzz.2022.3168050

[47] Mohammed RT, Zaidan AA, Yaakob R, Sharef NM, Abdullah RH, Zaidan BB, et al. Determining Importance of Many-Objective Optimisation Competitive Algorithms Evaluation Criteria Based on a Novel Fuzzy-Weighted Zero-Inconsistency Method. Int J Info Tech Dec Mak. 2021;21(01):195–241. doi: 10.1142/s0219622021500140

[48] Alamleh A, Albahri OS, Zaidan AA, Alamoodi AH, Albahri AS, Zaidan BB, et al. Multi-Attribute Decision-Making for Intrusion Detection Systems: A Systematic Review. Int J Info Tech Dec Mak. 2022;22(01):589–636. doi: 10.1142/s021962202230004x

[49] Haque AKMB, Bhushan B, Dhiman G. Conceptualizing smart city applications: Requirements, architecture, security issues, and emerging trends. Expert Systems. 2021;39(5). doi: 10.1111/exsy.12753

[50] Yas QM, Zaidan AA, Zaidan BB, Rahmatullah B, Abdul Karim H. Comprehensive insights into evaluation and benchmarking of real-time skin detectors: Review, open issues & challenges, and recommended solutions. Measurement. 2018;114:243–60. doi: 10.1016/j.measurement.2017.09.027

[51] Napi NM, Zaidan AA, Zaidan BB, Albahri OS, Alsalem MA, Albahri AS. Medical emergency triage and patient prioritisation in a telemedicine environment: a systematic review. Health Technol. 2019;9(5):679–700. doi: 10.1007/s12553-019-00357-w

[52] Yang Y, Wang H, Zhao Y, Zhang L, Li Y. Three-way decision approach for water ecological security evaluation and regulation coupled with VIKOR: A case study in Beijing-Tianjin-Hebei region. Journal of Cleaner Production. 2022;379:134666. doi: 10.1016/j.jclepro.2022.134666

[53] Song J, He Z, Jiang L, Liu Z, Leng X. Research on hybrid multi-attribute three-way group decision making based on improved VIKOR model. Mathematics. 2022;10(10). doi: 10.3390/math101010

[54] Gao Y, Li D, Zhong H. A novel target threat assessment method based on three-way decisions under intuitionistic fuzzy multi-attribute decision making environment. Engineering Applications of Artificial Intelligence. 2020;87:103276. doi: 10.1016/j.engappai.2019.103276

[55] Yao Y, Deng X. Sequential three-way decisions with probabilistic rough sets. In: IEEE 10th International Conference on Cognitive Informatics and Cognitive Computing (ICCI-CC’11). 2011. p. 120–5. doi: 10.1109/coginf.2011.6016129

[56] Khan MB, Deaconu AM, Tayyebi J, Spridon DE. Diamond Intuitionistic Fuzzy Sets and Their Applications. IEEE Access. 2024;12:176171–83. doi: 10.1109/access.2024.3502202

[57] Peng X, Yang Y. Fundamental Properties of Interval-Valued Pythagorean Fuzzy Aggregation Operators. Int J Intell Syst. 2015;31(5):444–87. doi: 10.1002/int.21790

[58] Menger K. Statistical Metrics. Proc Natl Acad Sci U S A. 1942;28(12):535–7. doi: 10.1073/pnas.28.12.535 16588583 PMC1078534

[59] Schweizer B, Sklar A. Probabilistic metric spaces. North-Holland, New York. 1983.

[60] Klement EP, Mesiár R, Pap E. Triangular norms. Dordrecht: Kluwer Academic Publishers; 2002.

[61] Klement EP, Mesiár R, Pap E. Triangular norms. Position paper I: basic analytical and algebraic properties. Fuzzy Sets and Systems. 2004;143(1):5–26.

[62] Klement EP, Mesiár R, Pap E. Triangular norms. Position paper III: continuous t-norms. Fuzzy Sets and Systems. 2004;145(3):439–54.

[63] Yang Y, Chin K, Ding H, Lv H, Li Y. Pythagorean fuzzy Bonferroni means based on T‐norm and its dual T‐conorm. Int J Intell Syst. 2019;34(6):1303–36. doi: 10.1002/int.22097

[64] Al-shami TM, Ibrahim HZ, Azzam AA, EL-Maghrabi AI. SR‐Fuzzy Sets and Their Weighted Aggregated Operators in Application to Decision‐Making. Journal of Function Spaces. 2022;2022(1):3653225.

[65] Al-shami TM. (2, 1)-fuzzy sets: properties, weighted aggregated operators and their applications to multi-criteria decision-making methods. Complex & Intelligent Systems. 2023;9(2):1687–705.

[66] Thakur SS, Banafar AS, Thakur M, Pandey Bajpai J, Prasad AK. Operations and Similarity Measures Between (m,n)-Fuzzy Sets. JIMS. 2024:21–39. doi: 10.22342/jims.30.1.1354.21-39

